# ROS-responsive drug delivery systems for chronic wound healing: advances, challenges, and translational perspectives

**DOI:** 10.1016/j.mmr.2026.100019

**Published:** 2026-04-04

**Authors:** Yuan Xiong, Qi-Peng Wu, Han Wang, Mohammad-Ali Shahbazi, Bo-Bin Mi

**Affiliations:** a Department of Orthopedics, Tongji Hospital, Tongji Medical College, Huazhong University of Science and Technology, Wuhan 430030, China; b Department of Orthopedics, Union Hospital, Tongji Medical College, Huazhong University of Science and Technology, Wuhan 430022, China; c Department of Anesthesiology, Peking University People’s Hospital; Department of Anesthesiology, Women and Children’s Hospital, Qingdao University, Qingdao 266000, Shandong, China; d Department of Biomaterials and Biomedical Technology, Personalized Medicine Research Institute (PRECISION), University Medical Center Groningen (UMCG), University of Groningen, Groningen 9713 AV, the Netherlands

**Keywords:** Chronic wounds, Reactive oxygen species (ROS), Drug delivery systems (DDS), Wound microenvironment, Translational medicine

## Abstract

Chronic wounds pose a substantial global health burden, due to the high incidence and recurrence rates, and associated morbidity. Excessive and sustained production of reactive oxygen species (ROS) is a hallmark of the chronic wound microenvironment, ultimately stalling the repair process. The pathological accumulation of ROS in chronic wounds has motivated the development of ROS-responsive drug delivery systems (DDS), which show considerable potential in improving wound healing outcomes. In this review, we provide a comprehensive overview of the advances in ROS‐responsive DDS for chronic wound healing, summarizing the design principles, material chemistry, and underlying ROS‐triggered functional mechanisms. Key translational challenges are discussed, including material biocompatibility, stability in protease‐ and ROS‐rich wound exudates, manufacturing scalability, and regulatory considerations. Finally, we outline future perspectives, emphasizing the integration of multi‐responsive functionalities, real‐time ROS monitoring, and advanced biomaterial engineering to accelerate clinical translation. By aligning therapeutic release with the dynamic redox status of chronic wounds, ROS‐responsive DDS holds considerable potential to redefine precision therapy for wound management.

## 1 Background

Chronic wounds represent a growing global health challenge [1]. Epidemiological studies suggest that approximately 1%–2% of the global population will experience a chronic wound during their lifetime [2], [3]. The prolonged treatment course, which typically involves repeated wound dressing care, systemic antibiotic administration, and other supportive therapies, results in substantial direct healthcare costs. In addition, high recurrence rates and frequent complications further increase the socio-economic burden. Together, these factors highlight the urgent need for more effective and innovative therapeutic strategies [4], [5].

Wound healing is a tightly orchestrated, multi-phasic process typically described in 4 overlapping stages: hemostasis, inflammation, proliferation, and remodeling [6], [7]. Immediately after injury, hemostasis involves platelet aggregation and fibrin clot formation to arrest bleeding and serve as a provisional matrix, with the release of growth factors that kick-start repair. The inflammatory phase follows as neutrophils and macrophages infiltrate the wound bed, clearing pathogens and debris, while secreting cytokines that orchestrate downstream reparative responses. Next, the proliferative stage is characterized by fibroblast proliferation, collagen deposition, angiogenesis, and re-epithelialization, forming robust granulation tissue. Lastly, during remodeling, collagen fibers are reorganized and matured, and tissue tensile strength is gradually restored [8], [9].

Reactive oxygen species (ROS) play context-dependent roles throughout wound healing. When maintained at low to moderate levels, ROS serve as signaling molecules that promote cell proliferation, angiogenesis, and host defense, facilitating effective tissue repair [10]. However, in chronic wounds, an imbalance in ROS homeostasis is evident. Persistent and excessive ROS generation induces oxidative damage to lipids, proteins, and nucleic acids, undermining cellular integrity and function [11]. Elevated ROS levels impair fibroblast proliferation, endothelial cell migration, and angiogenic signaling, while promoting matrix degradation and inhibiting collagen deposition, thereby arresting progress at the inflammatory stage or producing dysfunctional granulation tissue [12], [13].

**Fig. 1:**
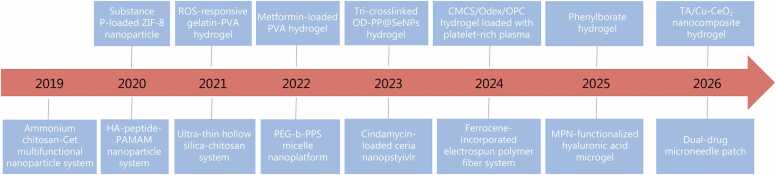
Generational overview of the ROS-responsive DDS in the management of chronic wound-healing during the past years. ROS. Reactive oxygen species; DDS. Drug delivery systems; ZIF-8. Zeolitic imidazolate framework-8; PVA. Poly(vinyl alcohol); CMCS. Carboxymethyl chitosan; Odex. Oxidized dextran; OPC. Oligomeric procyanidins; PEG-b-PPS. Poly(ethylene glycol)-block-poly(propylene sulfide); PAMAM. Poly(amidoamine).

Given these pathological characteristics, ROS present an attractive trigger for disease-targeted drug delivery systems (DDS). In this review, we systematically examine the development history of ROS-responsive DDS in the management of chronic wounds during the past years (Fig. 1). First, we introduce and categorize the primary ROS-responsive materials and platforms, highlighting their material chemistry and responsiveness mechanisms. Second, we analyze preclinical evidence detailing how these ROS-responsive DDS behave in chronic wound models, focusing on therapeutic efficacy, kinetics of ROS-triggered release, measures of healing, and mechanistic insights into how local redox modulation promotes wound repair. We then critically assess the challenges hindering clinical translation, such as material biocompatibility, stability in biologically complex wound exudates, manufacturing scalability, regulatory barriers, and user compliance issues. Finally, we project future opportunities by exploring the integration of smart functionalities to advance ROS-responsive DDS toward real-world applications for managing chronic wounds.

## 2 Pathophysiology of ROS in chronic wounds

Chronic wounds exhibit a distinctive ROS profile characterized by both elevated concentrations and dysregulated spatiotemporal distribution [14]. The predominant ROS species include superoxide anion radical (O_2_^•–^), hydrogen peroxide (H_2_O_2_), hydroxyl radicals (•OH), hypochlorous acid (HOCl), and peroxynitrite (ONOO^–^) [11]. Among these, O_2_^•–^ and H_2_O_2_ constitute the major contributors, with levels reported to be several-fold higher in chronic wounds than in acute wounds or healthy skin [15]. Elevated ROS production originates from diverse intracellular sources, including nicotinamide adenine dinucleotide phosphate (NADPH) oxidases (NOX2/NOX4) in neutrophils and macrophages, mitochondrial dysfunction in hypoxic or senescent cells, and sustained myeloperoxidase activity during chronic inflammation, with additional contributions from bacterial infection. These ROS are largely generated by activated immune cells and disturbed mitochondrial metabolism and are typically assessed using fluorescent probes, electrochemical methods, or biosensor-based platforms [16].

The intracellular localization of these ROS further contributes to cellular dysfunction. Mitochondrial accumulation of ROS impairs the generation of adenosine triphosphate and promotes mitochondrial permeability transition, whereas cytosolic ROS activates inflammatory transcription factors [17]. Excessive extracellular accumulation of ROS within the wound exudate also degrades matrix components and inactivates growth factors, collectively reinforcing the non-healing state [18].

**Fig. 2:**
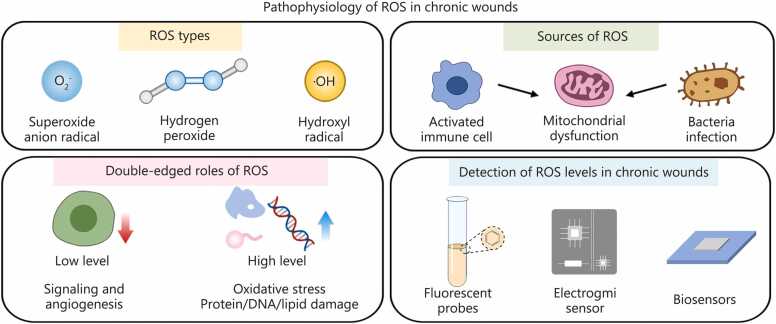
Pathophysiology of reactive oxygen species (ROS) in chronic wound healing. This section illustrates the major types and sources of ROS, highlights their dual roles in regulating or impairing the healing process, and summarizes approaches for detecting ROS levels in chronic wounds.

In cancer, ROS levels fluctuate dynamically and are buffered by enhanced antioxidant systems to support proliferative signaling [19]. Neurodegenerative diseases primarily involve mitochondrial oxidative injury within defined neuronal populations [20]. Cardiovascular disorders feature NOX-driven ROS surges that are episodic rather than persistent [21]. In contrast, chronic wounds display continuously high ROS levels across both intracellular and extracellular compartments, forming a persistent oxidative-inflammatory loop that lacks effective resolution mechanisms [22]. These distinct features make chronic wounds particularly amenable to ROS-responsive DDS that exploit pathological ROS elevation for selective activation (Fig. 2).

### 2.1 Sources of ROS

In chronic wounds, ROS are generated from multiple, often overlapping, sources [23]. Activated immune cells, particularly neutrophils and macrophages, recruited during persistent inflammation, produce ROS via NADPH oxidase-mediated oxidative bursts to kill invading pathogens [24]. Mitochondrial dysfunction in resident cells, such as fibroblasts and keratinocytes, also contributes to sustained ROS production, while impaired electron transport chain activity leads to electron leakage and excessive O_2_^•–^ generation [25].

Furthermore, several important enzymatic sources of ROS in non-immune cells also contribute substantially to oxidative dysregulation in chronic wounds. Xanthine oxidase highly active under ischemic and hypoxic conditions, produces O_2_^•–^ during purine metabolism and is markedly upregulated in diabetic and ischemic wounds [26]. Cyclooxygenases (COX-1/COX-2) and lipoxygenases, which participate in arachidonic acid metabolism, can generate ROS as by-products while amplifying inflammatory lipid mediator signaling, thereby exacerbating tissue injury, and delaying repair [27], [28]. Endothelial nitric oxide synthase, when uncoupled due to limited L-arginine or tetrahydrobiopterin, shifts from producing nitric oxide (NO) to generating O_2_^•–^, contributing to endothelial dysfunction and impaired angiogenesis [29]. Incorporating these non-immune enzymatic pathways may provide a more comprehensive understanding of ROS sources in chronic wound microeOS levels is essential for designing ROS-responsive DDS and monitoring redox dynamics in chronic wounds [30]. A variety of sensing platforms have been developed to detect distinct ROS species. Widely used small-molecule fluorescent probes, including 2’,7’-dichlorofluorescein diacetate (DCFH-DA) for general ROS [31], [32], hydroxyphenyl fluorescein (HPF) for •OH [33], dihydroethidium (DHE) or MitoSOX for O_2_^•–^ [34], [35], and boronate-based probes for H_2_O_2_ [36], primarily detect micromolar to sub-micromolar ROS concentrations. These sensors can be engineellular compartments, as DCFH-DA targets the cytosol, MitoSOX accumulates in mitochondria, and lysosome-targeted probes enable assessment of ROS in acidic organelles [37], [38]. Extracellular ROS can be monitored using cell-impermeable probes or electrochemical sensors designed to operate in wound exudate [39], [40].

In addition to fluorescent reporters, chemiluminescent probes and emerging nanoparticle (NP)-based sensors allow detection of specific ROS with enhanced sensitivity [41], [42]. Electrochemical ROS sensors embedded in wound dressings offer real-time, in situ monitoring of H_2_O_2_ and O_2_^•⁻^ in the wound microenvironment [43].

However, current ROS detection methods face significant limitations, as many probes exhibit poor selectivity due to cross-reactivity among ROS species, and some undergo auto-oxidation, generating false-positive signals [44]. Moreover, continuous ROS monitoring is constrained by photobleaching and limited temporal resolution, and probe performance is further compromised by pH variability, metal ions, and protease-rich wound exudates, leading to reduced detection accuracy in chronic wounds [44]. Importantly, most probes provide semi-quantitative rather than absolute ROS measurements, and in vivo quantification remains challenging due to attenuation of optical signals in tissue (Table 1) [45], [46], [47], [48], [49], [50], [51], [52], [53], [54], [55], [56], [57], [58], [59].

**Table 1:** Overview of ROS sensing modalities.

**Sensor type**	**Primary ROS detected**	**Typical cellular**	**Advantages**	**Limitations**	**References**
Fluorescent probes (DCFH-DA)	Broad ROS (H_2_O_2_, •OH, ONOO⁻)	Cytosol (after esterase cleavage)	Widely used; Simple imaging	Poor specificity; Auto-oxidation; PH-sensitive; Semi-quantitative	[45]
H_2_O_2_-specific probes (Boronate-based fluorophores, hyPer)	H_2_O_2_	Cytosol, nucleus, and targeted organelles	Higher specificity for H_2_O_2_; Genetically targetable	Signal saturation; Sensitive to pH; Limited use in protease-rich wounds	[46], [47]
Superoxide probes (DHE, MitoSOX red)	O_2_•⁻	Cytosol (dihydroethidium), mitochondria (MitoSOX)	Organelle-targetable; High ROS responsiveness	Cross-reactivity; Photo-oxidation; Limited quantification	[48], [49]
•OH probes (Hydroxyphenyl fluorescein, aminophenyl fluorescein)	•OH and some ONOO⁻	Cytosol/whole cell	High sensitivity to •OH	Not fully selective; Affected by metal ions	[50], [51]
HOCl probes (R19-S, MPO-specific indicators)	HOCl	Phagosomes, extracellular space	Measures neutrophil oxidative burst	Overlaps with other ROS/RNS; Rapid bleaching	[52], [53]
ONOO⁻ probes (Boronate-dye conjugates)	ONOO⁻	Cytosol	Higher selectivity for ONOO⁻	Reactivity with •OH causes false positives	[54]
Chemiluminescent probes (Luminol, lucigenin)	O_2_•⁻, H_2_O_2_	Whole wound tissue/exudate	High sensitivity; No excitation light needed	Low selectivity; Signal varies with pH and ions	[55], [56]
Electrochemical ROS sensors (H_2_O_2_ electrodes, graphene/Au nanoprobes)	H_2_O_2_, O_2_•⁻	Extracellular (wound fluid), wound dressings	Real-time, quantitative, continuous monitoring	Limited ROS specificity; Fouling in wound exudate	[57], [58], [59]

ROS. Reactive oxygen species; H₂O₂. Hydrogen peroxide; •OH. Hydroxyl radical; ONOO⁻. Peroxynitrite; O₂^•^⁻. Superoxide anion radical; HOCl. Hypochlorous acid; DCFH-DA. Dichlorodihydrofluorescein diacetate; hyPer. Hydrogen peroxide sensor; DHE. Dihydroethidium; MitoSOX. Mitochondria-superoxide indicator; R19-S. Rhodamine 19-sulfide; MPO. Myeloperoxidase; RNS. Reactive nitrogen species

## 3 Design principles of ROS-responsive DDS

**Fig. 3:**
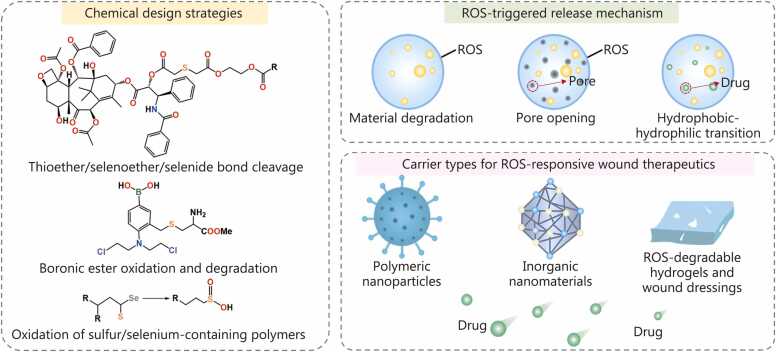
The schematic illustration of the chemical strategies and release mechanisms of reactive oxygen species (ROS)-responsive drug delivery systems (DDS).

ROS-responsive DDS for chronic wound therapy is engineered through rational chemical design, tailored release mechanisms, and strategic carrier selection (Fig. 3). Incorporation of ROS-cleavable linkages, such as thioether, selenoether, or boronic esters, enables selective responsiveness to oxidative stress, with selenium-containing bonds offering heightened sensitivity suitable for variable ROS levels in chronic wounds [60], [61], [62]. ROS-triggered release can be achieved via carrier degradation, nanopore opening, or hydrophobic-hydrophilic transitions, allowing precise modulation of release profiles. A broad spectrum of carriers, including polymeric NPs, inorganic nanomaterials, and ROS-degradable hydrogels, provides structural diversity and functional versatility [63], [64], [65]. Polymeric platforms offer tunable kinetics and enhanced biocompatibility, while inorganic systems enable multifunctional redox modulation. Hydrogels, particularly when integrated into wound dressings, combine localized delivery with moisture retention and potential ROS-sensing capability [66]. These design strategies lay the foundation for intelligent, site-specific therapies that align drug release with the oxidative dynamics of chronic wounds, thereby enhancing therapeutic efficacy and minimizing off-target effects.

Chronic wounds display heterogeneous pathophysiological characteristics and distinct ROS profiles, which require tailored design strategies for ROS-responsive DDS [67]. Diabetic foot ulcers are marked by sustained intracellular and extracellular ROS accumulation driven by chronic hyperglycemia and persistent inflammation. In this context, high ROS-threshold carriers, such as thioketal-based NPs or hydrogels, are particularly suitable for delivering angiogenic and anti-inflammatory therapeutics [67]. Pressure ulcers experience intermittent ischemia-reperfusion, resulting in transient ROS bursts [68]. Hence, rapid-response carriers, such as boronate-based micelles, are well suited to deliver antioxidants during acute oxidative peaks. Venous leg ulcers display sustained extracellular ROS accumulation, making long-acting, extracellular-targeted hydrogels or fibrous mats optimal for localized anti-inflammatory and pro-healing therapy [69]. Mixed or infected wounds may require multi-responsive DDS capable of sensing ROS alongside pH or enzymatic changes to coordinate antimicrobial, antioxidant, and regenerative functions [70]. This etiology-specific perspective emphasizes the importance of aligning carrier sensitivity, release kinetics, and payload distribution with the unique oxidative and cellular microenvironment of wounds, thereby maximizing therapeutic efficacy and safety.

As an ideal strategy for mild or early-stage wounds, low-threshold systems are designed to respond to lower concentrations of ROS (10–50 µmol/L), allowing for early intervention without premature drug release [11]. In contrast, high-threshold systems are better suited for more severe or infected wounds, where ROS levels can exceed 100 µmol/L. These DDS remain stable under normal oxidative stress and activate only when ROS concentrations reach a pathological threshold, thereby minimizing premature activation [71]. The main challenge in designing effective ROS-responsive DDS for wound healing lies in the variability of ROS levels in the wound microenvironment.

Moreover, although chronic and unresolved hypoxia in diabetic and chronic wounds is well recognized to impair endothelial function, reduce NO bioavailability, and hinder angiogenic sprouting, it is important to distinguish this pathological hypoxia from the transient or moderate hypoxia that naturally arises during early wound repair [22], [70]. Physiological hypoxia stabilizes HIF-1α, leading to the transcriptional activation of pro-angiogenic mediators such as vascular endothelial growth factor (VEGF), platelet-derived growth factor B (PDGF-B), and angiopoietin-2, which collectively promote endothelial proliferation, migration, and neovascularization. This adaptive response is essential for normal tissue regeneration [72]. However, in chronic wounds, persistent oxidative stress, inflammation, and metabolic dysfunction suppress HIF-1α signaling and disrupt this beneficial angiogenic cascade [72]. Recognizing this distinction underscores the need for DDS strategies that not only mitigate chronic hypoxia-induced dysfunction but also preserve or mimic the pro-regenerative hypoxic signaling required for effective vascular repair.

### 3.1 Chemical design strategies for ROS responsiveness

A variety of chemical bonds and moieties that respond to ROS have been reported (Table 2) [73], [74], [75], [76], [77], [78], [79], [80], [81], [82]. For instance, thioketal bonds exhibit high stability under physiological conditions but cleave rapidly under millimolar H_2_O_2_ or ONOO^–^ exposure, making them suitable for targeting severely oxidative wounds [83]. Boronate esters, by contrast, respond to lower micromolar H_2_O_2_ concentrations, offering higher sensitivity and faster response kinetics ideal for mild-to-moderate oxidative stress microenvironment [84]. Thioether and selenide/tellurium-based linkages show tunable redox sensitivity via heteroatom substitution, enabling gradated responsiveness across different ROS levels [85].

**Table 2:** Summary of major ROS-responsive chemical bonds and moieties.

**Functional group**	**Mechanism**	**Primary ROS trigger(s)**	**Typical sensitivity range**	**Key features**	**References**
Thioketal [-C(S)-C-]	Oxidative cleavage of C-S bonds forming ketones and sulfoxides	H_2_O_2_, •OH, ONOO⁻	High ROS (H_2_O_2_ 0.1–1.0 mmol/L)	High stability under physiological conditions; Effective in severely oxidative environments	[73], [74]
Thioether (R-S-R’)	Stepwise oxidation to sulfoxide/sulfone	H_2_O_2_, •OH	Moderate-to-high ROS (H_2_O_2_ 10–500 µmol/L)	Gradual oxidation allows tunable responsiveness; Suitable for moderate oxidative stress	[75]
Selenide/tellurium-based linkages	ROS-induced oxidation to selenoxide/telluroxide, triggering bond rearrangement or cleavage	H_2_O_2_, O_2_•⁻	Very low ROS (H_2_O_2_ 1–10 µmol/L)	Extremely high ROS sensitivity; Responsive under mild oxidative stress; Limited by potential cytotoxicity of heavy chalcogens	[76], [77]
Boronate ester	Nucleophilic substitution by H₂O₂ yielding phenol and boric acid derivatives	H_2_O_2_ (selective)	Low ROS (H₂O₂ 1–50 µmol/L)	Highly specific to H_2_O_2_; Fast kinetics; Suitable for precise, early-stage redox sensing and drug activation	[78]
Oxalate ester (-COO-COO-)	H_2_O_2_-mediated peroxy cleavage generating CO_2_ and alcohol	H_2_O_2_	Moderate ROS (H_2_O_2_ 10–200 µmol/L)	Enables gas-generation (bubble-assisted) and chemiluminescent responses; Applicable to imaging and on-demand release	[79]
Aryl ether	Oxidative elimination via aryl oxidation	HOCl, ONOO⁻	High ROS (H_2_O_2_>100 µmol/L)	Enhances selectivity toward specific ROS species in infected wounds	[80]
Proline or methionine residues in peptides	Oxidative cleavage or modification of side chains	H_2_O_2_, HOCl	Moderate ROS (H_2_O_2_ 10–100 µmol/L)	Natural, biocompatible ROS-sensitive motifs; Suitable for enzyme-linked systems	[79], [80]
Amino Borane/Boronic imidazolate coordination complexes	H_2_O_2_ oxidation breaks B-N or B-C bonds	H_2_O_2_	Low-moderate ROS (H_2_O_2_ 5–100 µmol/L)	Emerging hybrid materials with high selectivity and rapid kinetics; Promising for ROS-triggered nanocarriers	[81], [82]

ROS. Reactive oxygen species; H₂O₂. Hydrogen peroxide; •OH. Hydroxyl radical; ONOO⁻. Peroxynitrite; O₂^•^⁻. Superoxide anion radical; HOCl. Hypochlorous acid

A widely applied approach in ROS-responsive materials involves incorporating thioether, selenoether, or selenide linkages into the backbone or side chains of polymers. These bonds are selectively oxidized by elevated ROS levels to their corresponding sulfoxides/selenoxides or sulfones/selenones, leading to cleavage or conformational changes that trigger drug release [86]. Compared with thioethers, selenium-containing linkages are more redox-sensitive, enabling responsiveness to lower ROS concentrations, which is particularly beneficial for chronic wound environments with variable oxidative stress [87]. However, selenium has a narrow physiological window and excessive exposure can induce cytotoxicity [88]. In ROS-responsive DDS, selenium is typically incorporated at low molar ratios within polymer backbones or side chains, enabling selective oxidation and localized drug release at the wound site while minimizing systemic exposure [70]. Nonetheless, long-term safety and dose optimization remain critical considerations for clinical translation, and careful evaluation of selenium content, degradation products, and local accumulation is essential [70], [88].

A second strategy exploits boronic ester oxidation and degradation. Boronic esters and boronic acids are known to undergo rapid oxidative cleavage in the presence of H_2_O_2_, yielding phenols and boric acid derivatives [89]. This reaction is highly selective and can be tuned by structural modification of the boronic moiety [85]. In DDS, boronic esters may be incorporated as crosslinkers in polymer networks or as capping groups for mesoporous carriers, where ROS-induced cleavage results in carrier destabilization or gate opening [78].

A third approach utilizes sulfur- or selenium-containing polymers that undergo alterations of physicochemical properties upon oxidation, such as poly(propylene sulfide) or selenium-substituted analogs that transition from a hydrophobic to hydrophilic phase, causing polymer swelling, dissolution, or micelle disassembly [90], [91]. This transformation can release encapsulated hydrophobic drugs from micellar systems to modulate swelling and release kinetics of bulk materials, such as hydrogels, in response to local ROS levels.

### 3.2 ROS-triggered release mechanisms

ROS can activate drug release through several primary mechanisms [6], [92]. Carrier degradation occurs when ROS cleaves specific chemical bonds within the carrier, breaking the structure into soluble fragments that liberate the payload [93]. Nanopore opening is a common feature of inorganic and hybrid carriers, where ROS-sensitive gatekeepers block nanopores until exposed by oxidative cleavage, allowing drug diffusion [94], [95]. Hydrophobic-to-hydrophilic transitions of amphiphilic polymers or micelles occur when oxidation of hydrophobic segments increases polarity, destabilizing the core-shell structure of the carrier, thereby facilitating release [96], [97]. These mechanisms can operate singly or in combination, depending on the carrier type, desired release profile, and oxidative conditions of the target tissue.

### 3.3 Carrier types for ROS-responsive therapeutics

ROS-responsive carrier systems have been developed across diverse material platforms, each employing distinct activation mechanisms and offering unique therapeutic advantages (Table 3) [6], [92], [97], [98], [99], [100], [101], [102], [103], [104], [105], [106]. Polymeric NPs are among the most extensively studied carriers, with poly (lactic-co-glycolic acid) (PLGA), polyethylene glycol (PEG)-modified polymers, and block copolymers serving as versatile platforms [107], [108], [109]. Incorporation of ROS-cleavable linkages or pendant groups enables precise control over release kinetics in response to local oxidative stress, while PEGylation improves stability, circulation, and biocompatibility.

**Table 3:** Characteristics of representative ROS-responsive carrier systems and their characteristics.

**Type**	**Responsive moieties**	**Advantages**	**Limitations**	**References**
Polymeric nanoparticles	Thioketal, thioether, boronic ester	Excellent structural tunability; Stable in circulation; Precise control of degradation kinetics; High drug-loading capacity	Requires complex synthesis; Potential burst release at excessively high ROS; Degradation by-products must be biocompatible	[98]
Polymeric micelles	Thioether, selenide, boronate	Rapid, reversible response to low-moderate ROS; Suitable for hydrophobic drugs; Scalable self-assembly	Limited stability in protein-rich fluids; Possible premature disassembly; Small payload volume	[97]
Hydrogels	Thioketal, aryl boronate, oxalate	Excellent wound conformity; Prolonged local retention; Can serve as moist dressing with controlled release	Slower ROS diffusion may delay response; Potential incomplete degradation in low-ROS regions	[99]
Liposomal systems	Thioether, selenide, vinyl ether	Biocompatible and clinically familiar; Suitable for combined antioxidant-drug delivery	Membrane oxidation may compromise stability during storage; Limited control of release kinetics	[100]
Dendrimers and hyperbranched polymers	Boronate, thioketal, amino-borane	Precise molecular architecture; Multivalent surface modification; Good nucleic acid complexation	complex synthesis routes; Potential cytotoxicity at high generation numbers	[101], [102]
Inorganic-organic hybrid nanoparticles	MnO_2_, CeO_2_, Fe_3_O_4_ integrated with polymer shell	Dual function: ROS scavenging and stimulus-triggered release; Imaging capability	Possible long-term metal residue accumulation; Regulatory challenges for inorganic components	[103], [104], [105]
Electrospun fibrous films	Thioketal, boronate, hybrid nanocatalyst	Suitable for chronic wound dressings; Supports cell adhesion; Sustained and spatially localized release	Limited penetration depth; Mechanical fragility under wet conditions	[106]
Nanozymes	MnO_2_, Pt, CuO_2_, CeO_2_	Self-regulating redox balance; Combined therapeutic and diagnostic function	Requires careful dose and redox calibration; Potential oxidative toxicity at excess levels	[6], [92]

ROS. Reactive oxygen species; H₂O₂. Hydrogen peroxide; •OH. Hydroxyl radical; Nanozymes. Nanomaterial-based artificial enzymes

Inorganic nanomaterials offer structural tunability and multifunctionality. Metal-organic frameworks (MOFs) can be engineered with ROS-labile linkers or loaded with ROS-scavenging/producing agents, allowing synergistic redox modulation [92]. Mesoporous silica nanoparticles (MSNs) are frequently capped with ROS-degradable polymers or molecular gates to achieve “on-off” release [110], [111]. Additionally, nanozymes, nanomaterials with enzyme-like catalytic activity, can modulate ROS levels while simultaneously acting as delivery platforms, making them highly relevant to chronic wound microenvironments [112], [113], [114].

ROS-degradable hydrogels and wound dressings represent a particularly promising class for localized therapy. Hydrogels incorporating thioether or boronic ester crosslinks can degrade in oxidative environments and release embedded drugs directly into the wound bed [107], [115]. Integration of such hydrogels into wound patches enables sustained, site-specific therapy with the added benefit of maintaining a moist healing environment. Furthermore, these systems can be combined with ROS-sensing elements to create “smart” dressings capable of both monitoring and responding to the oxidative status of the wound [115].

Notably, the cleavage of thioether linkages typically generates sulfoxide or sulfone species, which generally have low toxicity and are readily metabolized through oxidative pathways in vivo. Since excessive oxidation may alter local redox balance, thioether-containing systems are usually applied at controlled doses [116]. Similarly, boronic ester or boronate linkages degrade into phenolic derivatives and boric acid, which exhibit both good biocompatibility and rapid renal clearance at therapeutic concentrations [117].

## 4 Therapeutic applications in chronic wound healing

### 4.1 Antimicrobial therapy

The management of bacterial infection of chronic wounds, particularly diabetic ulcers, remains a cornerstone of therapeutic strategies due to the high risk of persistent biofilm formation, antibiotic resistance, and exacerbated inflammatory responses [85]. Conventional systemic antibiotics often fail to eradicate pathogens within the dense extracellular polymeric matrix of biofilms, while prolonged usage contributes to antimicrobial resistance [85], [92]. Advanced biomaterial-based approaches now offer targeted, sustained, and microenvironment-responsive antimicrobial activity, often combined with complementary functionalities, such as oxygenation, ROS regulation, and immune modulation [118], [119]. For instance, a programmed *Haematococcus pluvialis* (HEA)-based hydrogel system demonstrated light intensity-dependent functionalities, where high-intensity irradiation (658 nm) induced potent photothermal antibacterial activity capable of disinfecting wound surfaces, while lower light intensities activated photosynthetic oxygen release to support vascular regeneration [120]. Notably, this dynamic platform enabled sequential modulation of infection, hypoxia, and oxidative stress, resulting in accelerated healing of infected diabetic wounds in vivo. Similarly, a bilayer hydrogel incorporating *Chlamydomonas reinhardtii* microrobots in the lower layer for bio-oxygenation and silver-fulvic acid (FA) NPs in the upper layer for photothermal bacterial eradication achieved dual action against biofilms and hypoxia, representing an integrated “three-pronged strategy” for management of chronic diabetic wounds [121].

Recent innovations in antimicrobial therapy for chronic wounds also focus on constructing multifunctional catalytic and supramolecular systems to disrupt bacterial metabolism and quorum sensing while simultaneously enhancing wound repair [103], [105]. In one study, a hyperbranched poly-L-lysine (HBPL)-crosslinked hydrogel loaded with MnO_2_ nanozymes (HMP hydrogel) displayed broad-spectrum bactericidal activity against methicillin-resistant *Staphylococcus aureus* (*S. aureus*), *Escherichia coli* (*E. coli*)*,* and *Pseudomonas aeruginosa*, with killing efficiencies exceeding 94% even at high bacterial loads (10^9^ CFU/ml) [102]. Beyond direct killing, HBPL inhibited bacterial quorum sensing and downregulated virulence gene expression, thereby impairing bacterial communication and biofilm maturation. Such combined antibacterial and quorum-quenching properties resulted in reduced neutrophil infiltration, increased M2 macrophage polarization, and enhanced angiogenesis in infected diabetic wound models. Another example is the guanosine-driven hyaluronic acid (HA) supramolecular hydrogel, which harnessed guanosine self-assembly into G-quartets to load hemin with peroxidase-like activity, enabling localized •OH generation in the presence of H_2_O_2_ [122]. Incorporation of glucose oxidase further modulated the wound glucose microenvironment and augmented antibacterial effects, while dynamic crosslinking provided adaptability to the wound’s biochemical milieu. These supramolecular and enzymatic strategies underscore the shift from static antimicrobial coatings toward responsive, multifunctional wound dressings capable of counteracting microbial persistence while supporting host tissue recovery.

Balancing antimicrobial potency with preservation of host tissue integrity has become a critical focus of chronic wound therapy, as excessive ROS-mediated bacterial killing can exacerbate inflammation and impede regeneration [102]. To address this issue, redox-modulatory antimicrobial platforms have been developed to fine-tune oxidative stress within the wound bed. A phytochemical-nanozyme hybrid utilized Ce^3^⁺/Ce^4^⁺ cycling and ferulic acid radical scavenging to exert controlled antibacterial effects against *S. aureus* and *E. coli,* while activating the nuclear factor erythroid 2-related factor 2 (Nrf2)/heme oxygenase-1 (HO-1) antioxidant pathway to promote angiogenesis and collagen deposition [123]. Likewise, a sono-piezodynamic therapy-enabled nanocatalytic membrane composed of black phosphorus/V_2_C MXene heterojunctions achieved clearance of drug-resistant bacteria under ultrasound-triggered ROS generation, followed by ROS scavenging when stimulation ceased, thereby preventing excessive oxidative damage [96]. Localized delivery approaches, such as copper-manganese oxide-loaded microneedles, have further demonstrated effective killing of bacteria, ROS scavenging, and regulation of macrophage polarization through combined enzymatic and photothermal mechanisms [119]. Collectively, these advances highlight a paradigm shift in chronic wound antimicrobial therapy from singular bactericidal modalities toward spatiotemporally controlled, microenvironment-adaptive systems that couple infection control with regenerative support.

The management of bacterial infection in chronic wounds, particularly in diabetic ulcers, remains a cornerstone of therapeutic strategies due to the high risk of persistent biofilm formation, antibiotic resistance, and exacerbated inflammatory responses [67]. Biofilms, formed by bacterial colonies encased in an extracellular polymeric matrix, protect the pathogens from host immune cells and antibiotics, making infections extremely difficult to eradicate [124]. Moreover, the prolonged use of antibiotics to combat these infections often leads to the development of antibiotic resistance, further complicating treatment [125]. Recent studies highlight that biofilm-associated infections in diabetic ulcers not only increase the risk of chronic inflammation but also delay wound healing by hindering normal immune response and tissue regeneration [126], [127]. These challenges underscore the need for advanced antimicrobial strategies, such as ROS-responsive DDS, which can target bacterial biofilms and modulate the wound microenvironment to improve healing outcomes.

ROS-responsive DDS for antimicrobial therapy represents a promising approach to overcome the persistent challenge of bacterial infections in chronic wounds, particularly in diabetic ulcers [70]. These systems utilize the inherent oxidative stress of the wound environment to release therapeutic agents in a controlled manner, ensuring targeted and sustained antimicrobial activity [96]. The combination of antimicrobial properties with ROS regulation is critical, as it reduces the risk of excessive ROS buildup, which could impair healing [67]. Moreover, photothermal and catalytic strategies illustrate the dual functionality of these systems, providing both infection control and wound regeneration. The ability to simultaneously modulate infection, inflammation, and hypoxia represents a shift toward multifunctional platforms, where the synergy between ROS and therapeutic payloads drives more effective wound healing outcomes [102], [104].

### 4.2 Anti-inflammatory modulation

Recent studies have demonstrated that incorporating ROS-responsive or antioxidative components into wound dressings enables precise modulation of the inflammatory phase [128], [129], [130]. For instance, a double network hydrogel, combining a gelatin-methacrylate and copper-alginate network with luteolin-loaded NPs (GSC/PBE@Lut), achieved spatiotemporal control over the release of luteolin, a flavonoid with potent anti-inflammatory properties, by exploiting both pH and ROS responsiveness [129]. This system promoted the polarization of macrophages toward the anti-inflammatory (M2) phenotype, while simultaneously attenuating the proinflammatory (M1) response, thereby accelerating the resolution of inflammation. Similarly, a dual-component particulate polyacrylic acid/madecassoside dressing facilitated robust microenvironmental modulation by downregulating inflammatory cytokines and steering macrophage polarization, which synergistically enhanced tissue regeneration [128]. These approaches underscore the importance of integrating immunomodulatory functions directly into wound dressings to actively guide the inflammatory phase toward a regenerative trend.

Beyond passive anti-inflammatory effects, some DDS have been engineered to actively target specific molecular pathways implicated in inflammation [131], [132]. The glucose-responsive HA-phenylboronic acid (PBA)-FA/EN106 hydrogel combines HA, PBA, and FA to create a glucose-responsive, antibacterial, and antioxidative environment that promotes healing. The “EN106” component is released in a glucose-dependent manner, stimulating angiogenesis and reducing oxidative stress to accelerate wound repair [131]. The HA-PBA-FA/EN106 hydrogel exemplifies such precise intervention, where controlled release of the fem-1 homolog b-folliculin-interacting protein 1 axis inhibitor mitigated mitochondrial ROS overproduction and rescued angiogenic dysfunction under hyperglycemic conditions [131]. By simultaneously delivering FA, an agent with intrinsic anti-inflammatory and antibacterial properties, this platform provided multifaceted microenvironmental regulation, breaking the cycle of OS and inflammation [131]. Similarly, ROS-responsive hydrogel matrices embedding lipid NPs for co-delivery of antimicrobial peptides and puerarin effectively suppressed the bacterial burden and modulated vascular inflammation [100]. In another innovative example, a multifunctional microneedle system (CeO₂@Tau@Hydrogel@Microneedle) combined deep-tissue antioxidant delivery with direct inhibition of the ROS/NF-κB axis in macrophages, dampening proinflammatory signaling cascades and preventing chronic immune activation [103]. The spatial precision afforded by microneedle-based penetration ensures that anti-inflammatory agents reach deeper tissue layers where persistent inflammatory cells reside, improving the therapeutic outcomes in otherwise refractory chronic wounds.

A further evolution of ROS-targeted anti-inflammatory DDS is the ability to sequentially and adaptively regulate inflammation in concert with other wound healing phases [133]. This is exemplified by dissolvable dual-layer microneedles incorporating selenium-doped carbon quantum dots for immediate ROS scavenging, followed by sustained release of astilbin to promote M2 macrophage polarization and angiogenesis [132]. Such staged release aligns with the physiological transition from inflammation to proliferation, but reduced early suppression of inflammation, which could hinder pathogen clearance. The multifunctional dihydromyricetin-loaded Pluronic F-127-based hydrogel extends this principle by integrating glycemic control with anti-inflammatory activity, addressing systemic drivers of chronic inflammation in diabetic wounds [133]. Importantly, these platforms combine sustained release kinetics, antioxidant capacity, and macrophage reprogramming, creating a synergistic effect that overcomes the limitations of monotherapy. Collectively, these findings highlight that anti-inflammatory modulation in chronic wound healing is most effective when achieved through multifunctional, ROS-responsive DDS that integrate temporal control, microenvironmental targeting, and combinatorial therapy.

Anti-inflammatory modulation in chronic wound healing is essential to overcome prolonged inflammation and facilitate tissue regeneration. ROS-responsive DDS can fine-tune the wound microenvironment by spatiotemporally controlling the release of anti-inflammatory agents. Systems, such as the GSC/PBE@Lut hydrogel, demonstrate how ROS and pH responsiveness can direct macrophage polarization toward the anti-inflammatory M2 phenotype to accelerate tissue repair. Moreover, these platforms not only mitigate inflammation but also engage with key molecular pathways to precisely modulate mitochondrial ROS production, fostering a regenerative microenvironment [129]. Multifunctional DDS that integrates antioxidant properties with anti-inflammatory interventions are particularly promising by enabling precise, dynamic regulation of the inflammatory response, while preserving immune function and accelerating wound healing.

### 4.3 Angiogenesis and tissue regeneration

Angiogenesis, the sprouting of new blood vessels from the pre-existing vasculature, is indispensable for effective chronic wound repair by ensuring adequate oxygen and nutrient delivery to support tissue regeneration [134]. In chronic wounds, persistent inflammation, pathological ROS accumulation, and hypoxia synergistically impair endothelial proliferation, vessel maturation, and microvascular stability, ultimately delaying or preventing closure [7]. ROS-based DDS have emerged as powerful tools to address these obstacles, enabling precise modulation of the wound microenvironment through targeted oxidative stress regulation, staged release of pro-angiogenic factors, and combination with oxygen delivery or antimicrobial strategies.

Recent biomaterial advances have demonstrated that angiogenesis is most effective when preceded by the resolution of inflammation and normalization of ROS levels. For example, bilayer alginate hydrogels encapsulating polyelectrolyte complex NPs co-loaded with interleukin (IL)-10 and angiogenic growth factors exhibit a temporally programmed release, first releasing IL-10 to suppress inflammation via janus kinase 1/signal transducer and activator of transcription 3 activation, followed by VEGF/PDGF to stimulate vessel sprouting and stabilization in diabetic murine wounds [135]. By using ROS-labile linkages or scavenging moieties within the hydrogel network, such systems can maintain VEGF bioactivity in oxidative environments and prevent premature degradation. Similarly, inflammation- and ROS-responsive hydrogels incorporating Prussian blue NPs with VEGF have been shown to quench excess ROS production, preserve growth factor function, and significantly enhance capillary density in type 2 diabetic wound models [136].

ROS-responsive DDS integration also benefits targeting molecular suppressors of neovascularization [137]. Grancalcin, a proinflammatory protein elevated in diabetic wounds, inhibits angiogenesis via transient receptor potential melastatin 8 channel signaling [138]. Sustained delivery of grancalcin-neutralizing antibodies from gelatin methacrylamide hydrogels restores endothelial activity and vessel formation. When combined with ROS-scavenging components, these hydrogels further relieve oxidative endothelial injury, amplifying angiogenic outcomes [138]. Likewise, ROS-controlled oxygen delivery systems such as oxygen-generating microspheres embedded in ROS-responsive hydrogels, simultaneously mitigate hypoxia and oxidative stress, while enhancing endogenous expression of angiogenic growth factors [139]. Notably, these oxygenation platforms achieve robust vascular network formation without exogenous pharmacologics, highlighting the efficacy of microenvironmental reprogramming through redox balance restoration.

The integration of extracellular vehicles (EVs) into ROS-adaptive dressings has expanded pro-angiogenic therapy toward more targeted and sustained outcomes [140], [141]. EVs from *Lactobacillus rhamnosus* GG (LGG) promote angiogenesis and epithelialization via miR-21-5p-mediated metabolic reprogramming of endothelial and epithelial cells [140]. When incorporated into ROS-scavenging hydrogels, EV bioactivity is preserved under oxidative stress, prolonging their pro-healing effect. Similarly, glycoengineered EVs modified with sialyl Lewis X (sLeX), encapsulated in antibacterial Gel/PL-5 hydrogels, exhibit E-selectin-mediated targeting to inflamed vasculature, accelerating vascular regeneration in infected diabetic wounds [141]. Multifunctional scaffolds embedding adipose-derived stem cells with angiogenic cues such as ginsenoside RG1 and stromal cell-derived factor-1 also demonstrate synergistic neovascularization and neurogenesis, especially when ROS-responsive matrices protect cell viability and modulate local redox homeostasis [142].

Catalytic and enzymatic platforms further illustrate the versatility of ROS-based DDS in coupling infection control with angiogenesis promotion. For instance, glucose oxidase-functionalized copper metal-organic framework (Cu-MOF/GOX) hydrogels consume excess glucose, a metabolic driver of oxidative stress, while generating H_2_O_2_ and NO in situ, the latter acting as a potent vasodilator and pro-angiogenic mediator [143]. Similarly, platinum-armed Fe-based MOF nanozymes (Pt@FeMOF) embedded in ROS-adaptive cryogels achieve rapid hemostasis, eradicate biofilms via synergistic Fenton chemistry, and relieve oxidative stress-induced endothelial senescence, thereby restoring angiogenic capacity [144]. Collectively, these examples emphasize that effective angiogenesis in chronic wounds requires more than isolated growth factor delivery, but also demands multi-pronged, ROS-aware strategies that integrate redox regulation, inflammation resolution, hypoxia mitigation, and infection control.

Angiogenesis plays a central role in wound healing by ensuring an adequate supply of oxygen and nutrients to the regenerating tissue [67], [96]. ROS-responsive DDS provides a mechanism for spatiotemporally controlled release of pro-angiogenic factors, such as VEGF and IL-10, while simultaneously modulating oxidative stress to enhance endothelial function and capillary growth [145]. Systems like bilayer alginate hydrogels and Prussian blue NP-based hydrogels demonstrate how ROS scavenging can preserve the bioactivity of growth factors and improve vascular network formation in diabetic wound models [136]. Furthermore, the integration of EVs into ROS-responsive hydrogels exemplifies how cell-based therapies can be incorporated into these systems to enhance tissue regeneration [115]. The multifunctionality of these DDS, combining angiogenesis promotion with oxidative stress management and immune regulation, sets them apart as effective therapeutic options for chronic wound healing.

### 4.4 Multimodal therapy

Chronic wound healing demands therapeutic strategies that address multiple pathological factors in parallel, persistent infection, excessive inflammation, oxidative stress, impaired angiogenesis, and deficits in tissue regeneration [29], [70], [134]. In this context, ROS-based multimodal DDS offer a unique advantage: they not only act as carriers for diverse therapeutic agents but also actively participate in microenvironmental regulation by scavenging excess ROS or harnessing ROS-triggered release mechanisms [146], [147]. This dual capability allows integration of antibacterial, immunomodulatory, pro-angiogenic, and ECM remodeling functions within a single therapeutic platform, achieving superior efficacy compared to monotherapies.

A representative example is the prodigiosin-loaded SN-PB@PG nanocomplex for bacterial-infected chronic wounds [148]. This hybrid system couples sodium nitroprusside with prussian blue NPs, the latter functioning as both ROS scavengers and photothermal agents, followed by loading with the antibacterial pigment prodigiosin (PG). Upon near-infrared (NIR) irradiation, Prussian blue NPs generate mild hyperthermia, which not only enhances biofilm disruption but also facilitates on-demand release of NO and PG [148]. Beyond antimicrobial activities, NO promotes angiogenesis via VEGF and CD31 upregulation, while PG eradicates biofilms and suppresses proliferation of multidrug-resistant pathogens [148]. In diabetic wound models, this ROS-adaptive platform achieved wound area reduction to 10.6% by day 11 and improved flap survival rates, illustrating how photothermal-assisted, gas-mediated, and ROS-regulated release can simultaneously control infection and restore vascular networks [148].

Another notable ROS-related approach employs ferrocene-HA organic copolymer (FHoC) hydrogels as multifunctional dressings [149]. The inherent redox activity of ferrocene enables modulation of ROS levels at the wound site to reduce OS-driven tissue damage. Fe²⁺ ions released from ferrocene not only participate in mild ROS regulation but also enhance angiogenesis [149]. The HA backbone provides biocompatibility and moisture balance, while the microcrystalline, hydrophobic domains yield exceptional exudate absorption (up to 150-fold of dry weight) without structural collapse [149]. This combination of physical wound protection, oxidative stress modulation, and pro-angiogenic stimulation addresses the common chronic wound issue of excessive exudate, inflammation, and delayed granulation tissue formation, while also offering a scaffold for localized drug or cell delivery.

ROS regulation also plays a key role in biofilm eradication and long-term infection control. Polyurea-based multimodal interaction nanogels employ multiple intermolecular interactions for high-capacity antimicrobial loading, including quorum-sensing inhibitors (QSI) [150]. By disrupting bacterial communication pathways, QSIs prevent recolonization, while antibiotics provide immediate biofilm clearance [150]. When engineered with ROS-responsive elements, such nanogels can release antimicrobials in oxidative infection niches, ensuring localized, on-demand therapy [150]. Similarly, photothermal hydrogels incorporating bacteria-capturing bio-MOFs (QCSMOF-Van) combine vancomycin delivery with ROS-mediated metabolic disruption via Zn²⁺ release, while shifting macrophages toward the pro-regenerative M2 phenotype [146]. The result is a coordinated antibacterial, immunomodulatory, and angiogenic response in complex wound environments.

A further dimension involves immune reprogramming alongside oxidative stress regulation. Self-adapting biomass hydrogels based on carboxymethyl chitosan have been engineered to co-deliver antibacterial peptides and miR-301a-loaded NPs within a ROS-sensitive matrix [151]. This design enables peptide release in oxidative microenvironments, providing sustained clearance of Gram-positive and -negative bacteria, while miR-301a reprograms macrophages toward the anti-inflammatory M2 phenotype via the phosphatase and tensin homolog/phosphoinositide 3-kinase gamma/mammalian target of rapamycin pathway [151]. By attenuating ROS-driven chronic inflammation and simultaneously controlling infection, these hydrogels create a permissive environment for tissue regeneration [151]. Taken together, ROS-based multimodal DDS represent a significant shift from single-target interventions to integrated, redox-aware therapeutic platforms by synchronizing antimicrobial, anti-inflammatory, pro-angiogenic, and regenerative actions in a spatially and temporally controlled manner to address the multifactorial pathology at the biochemical root of chronic wounds.

While we primarily emphasize ROS-responsive DDS designed for exogenous drug delivery, it is indeed important to acknowledge that ROS-responsive materials can also act as bioactive regulators of endogenous repair mechanisms, even in the absence of external therapeutic agents [8], [70]. These materials function through intrinsic ROS-scavenging activity, catalytic redox regulation, or fine-tuning of redox-associated signaling pathways, contributing to the restoration of redox homeostasis and subsequent tissue regeneration [70], [152]. For instance, nanozymes and ROS-scavenging polymers can neutralize excessive ROS, mitigate oxidative stress and inflammation while enhancing fibroblast proliferation and angiogenesis [112]. Similarly, ROS-degradable hydrogels not only provide a protective, moist environment but also dynamically remodel in response to oxidative cues, facilitating cell migration and matrix deposition [153]. Furthermore, ROS-tunable scaffolds can modulate intracellular redox-sensitive pathways such as Nrf2/antioxidant response element and HIF-1α, thereby stimulating endogenous antioxidant and angiogenic responses [154]. Integrating these self-regulatory, material-intrinsic effects with drug delivery functions represents a promising direction for developing next-generation ROS-responsive biomaterials that both deliver exogenous therapeutics and reprogram endogenous wound repair processes.

## 5 Theranostic platforms for chronic wounds

The integration of real-time diagnosis and on-demand therapy, theranostics, represents a paradigm shift in chronic wound management, enabling personalized interventions tailored to the evolving wound microenvironment [155]. In ROS-responsive DDS, theranostic platforms extend beyond ROS neutralization by using aberrantly elevated ROS as a local trigger for therapeutic payload release and concurrent in situ monitoring.

As a key differentiator, theranostic platforms integrate diagnostic capability with therapeutic functions [156]. For example, a porous silicon (Psi)-based carrier encapsulating an NIR-active dye (IR820) in a calcium-sealed form (I-CaPSi) is an innovative theranostic approach that uses ROS exposure to modulate the photothermal signal. Upon oxidative degradation of the Psi framework, IR820 is released, reducing photothermal output, which is then detected non-invasively using a smartphone-based thermal camera, providing a real-time wound monitoring system [157]. This enables continuous assessment of wound status while simultaneously delivering photothermal and photodynamic antibacterial effects and promoting fibroblast migration and angiogenesis via NIR irradiation. This combined approach provides both diagnostic and therapeutic functions in a single, user-friendly platform for chronic wound theranostics [157].

Furthermore, ROS-responsive smart dressings represent another important theranostic development [158]. These dressings integrate ROS-sensitive sensors with therapeutic release capabilities, enabling continuous tracking of wound biomarkers without the need to remove the dressing [159]. These sensors can activate closed-loop or externally triggered release of ROS scavengers, antimicrobials, or pro-angiogenic factors, directly responding to real-time wound conditions and thus providing dynamic therapeutic feedback [155]. The key innovation of these platforms is the combination of diagnostic monitoring with therapeutic action in real-time, enabling personalized treatment adjustments.

Another promising example of theranostics for chronic wounds involves nanomedicine-based systems. For instance, trisulfide-derived lipid NPs encapsulating *IL-4* mRNA can target ROS in the wound environment, enabling the NPs to act both as ROS scavengers and as delivery vehicles for therapeutic mRNA. Upon internalization by macrophages, *IL-4* mRNA is translated to shift macrophages from the proinflammatory M1 phenotype to the pro-regenerative M2 phenotype, which accelerates tissue repair [160]. Coupled with appropriate imaging modalities, such as ROS-activated fluorescent probes or thermal signal transduction, this system not only facilitates therapeutic release but also allows for molecular-level monitoring of both ROS dynamics and therapeutic progress [161]. These examples illustrate that theranostic platforms extend beyond multimodal therapy by integrating diagnostic sensors with responsive therapeutic actions, thus providing real-time feedback and control over wound healing.

## 6 Translational considerations and future perspectives

ROS-responsive DDS offers unique advantages in the treatment of chronic wounds, especially in conditions like diabetic ulcers, where ROS accumulation is a hallmark of the wound microenvironment. These systems exploit localized oxidative stress to enable targeted drug release at the wound site, minimizing systemic side effects and ensuring that therapeutic drug concentrations are maintained in the affected area [100]. However, the variability in ROS levels across different wound types presents a challenge to the consistent performance of ROS-responsive DDS, as fluctuations in ROS levels can affect their effectiveness in achieving optimal drug release [161]. In contrast, other stimuli-responsive DDS, such as those triggered by pH, enzymes, or temperature, have also been applied for chronic wound healing [70]. For certain infected or necrotic tissues, pH-responsive systems can release drugs in acidic environments, but lack the specificity of ROS-responsive systems, and the pH in chronic wounds may fluctuate significantly [127], [145]. Enzyme-responsive DDS, targeting specific proteases like matrix metalloproteinases, can effectively promote tissue remodeling, but applicability may be limited depending on the wound types [6]. Temperature-responsive systems, which rely on changes in local temperature, may face challenges in chronic wounds due to the lack of consistent temperature variation [70]. While alternative stimuli-responsive DDS offer complementary therapeutic approaches, ROS-responsive systems stand out as highly specific for the unique microenvironment of chronic wounds, demonstrating superior potential in clinical applications [6], [60].

Chronic oxidative stress also plays a significant role in driving pathological fibrosis during impaired wound healing, an aspect increasingly recognized as a major barrier to functional tissue regeneration [23], [24]. Persistent ROS accumulation activates fibroblasts and myofibroblasts through pathways such as transforming growth factor beta/smad, mitogen-activated protein kinase, and Wnt/β-catenin, leading to excessive ECM deposition and aberrant collagen cross-linking [162]. In chronic wounds, this sustained profibrotic signaling disrupts the balance between ECM synthesis and degradation, resulting in prolonged granulation tissue persistence, stiffened wound beds, and impaired re-epithelialization [163]. Furthermore, ROS-induced oxidative modification of matrix proteins alters their biomechanical properties and perpetuates fibroblast activation in a feed-forward manner [164]. By enabling controlled ROS scavenging and microenvironment modulation, ROS-responsive DDS offer a promising strategy to attenuate fibroblast hyperactivation, normalize ECM remodeling, and reduce fibrosis-related wound chronicity [165]. Incorporating antifibrotic capabilities into ROS-responsive platforms may therefore enhance both structural and functional recovery in chronic wound healing.

Emerging living materials, including living therapeutic materials and living biotherapeutics, offer a promising avenue for chronic wound healing and can be integrated with redox-regulated or ROS-responsive strategies [166]. For example, living hydrogels encapsulating *Lactobacillus rhamnosus* engineered with intracellular nano-selenium combine antimicrobial activity with ROS scavenging, modulating the inflammatory microenvironment, and promoting repair [167]. Such systems dynamically adapt to the wound milieu, with embedded cells or microbes acting as in situ redox regulators by producing antioxidant enzymes, secreting ROS scavengers, or releasing therapeutic payloads in response to oxidative cues 175]. ROS levels can further modulate microbial metabolism, enabling self-regulation of therapeutic activity. Hybrid systems that integrate living materials with synthetic ROS-responsive scaffolds could combine the adaptive, self-renewing properties of living cells with precise release kinetics of engineered polymers, offering sustained, site-specific therapy [168]. Successful translation of these systems requires careful design to balance safety, metabolic load, and responsiveness to pathological ROS, highlighting the potential and challenges of living therapeutics in chronic wound management.

The clinical translation of ROS-based DDS for chronic wound therapy hinges first and foremost on ensuring material safety and biocompatibility [70]. Many current ROS-responsive platforms employ inorganic NPs, MOFs, or organometallic components, which, while effective in laboratory studies, may raise concerns regarding systemic toxicity, metal ion accumulation, and potential off-target redox reactions in human tissues [169], [170]. Even naturally derived polymers, such as chitosan or HA, require careful modification and thorough evaluation to avoid eliciting immune hypersensitivity or interfering with normal tissue homeostasis [171], [172], [173]. Regulatory agencies will require detailed toxicological profiling, encompassing not only acute and subchronic toxicity but also long-term biodistribution, clearance pathways, and effects on distant organs. Achieving a balance between potent ROS reactivity and physiological safety is thus a central challenge, necessitating iterative material design, surface functionalization to minimize nonspecific interactions, and the incorporation of biodegradable linkages that allow safe metabolic elimination [14].

Beyond safety, the long-term stability and storage conditions of ROS-based DDS remain a critical hurdle for real-world deployment. Many ROS-responsive carriers rely on labile chemical bonds, catalytic NPs, or sensitive biological cargos, which may degrade or lose functionality under ambient conditions [105], [174]. Moisture, oxygen, and light exposure can prematurely trigger ROS-scavenging or ROS-generating reactions, depleting therapeutic efficacy before application. For hospital and field settings, particularly in low-resource regions, wound dressings should be stable at room temperature for extended periods, packaged to prevent premature activation, and compatible with sterilization methods such as gamma irradiation or ethylene oxide treatment [67]. Achieving this requires advances in encapsulation strategies and the use of protective coatings or secondary shells that preserve reactivity until contact with the wound microenvironment. Addressing these storage and stability concerns is essential to ensure that ROS-based systems maintain consistent therapeutic performance across diverse clinical scenarios.

Achieving scalable manufacturing while preserving product quality and cost efficiency remains a major translational challenge. Laboratory protocols for synthesizing ROS-responsive nanomaterials often involve multi-step reactions, organic solvents, or specialized equipment that are not readily transferable to large-scale manufacturing [175], [176]. Batch-to-batch variability can significantly impact ROS sensitivity, release kinetics, and safety profiles, complicating regulatory approval. Cost considerations are particularly pressing for chronic wound care, where treatments may need to be applied repeatedly over weeks to months [177]. To be commercially viable, production pipelines should integrate high-throughput synthesis, green chemistry approaches, and robust quality control systems. Furthermore, discrepancies between preclinical animal models and human wound microenvironments represent a substantial translational barrier [178]. Common diabetic or ischemic wound models in rodents differ from human wounds in size, chronicity, microbial diversity, and immune responses, leading to overestimation of therapeutic efficacy in early studies [70], [179]. Incorporating large-animal models and ex vivo human skin cultures, alongside standardized outcome measures, will improve predictive accuracy and accelerate regulatory acceptance.

In the future, the next generation of ROS-responsive systems for chronic wound therapy will likely move toward personalized and precision medicine [180], [181]. By profiling an individual patient’s wound ROS spectrum, using minimally invasive biosensors, clinicians could select or tune DDS formulations to match the oxidative stress burden and healing phase, avoiding both under- and over-scavenging of ROS [43]. Integration with wearable electronics offers a powerful avenue for continuous ROS monitoring and closed-loop feedback control, where real-time data could trigger on-demand drug release, photothermal activation, or oxygen generation [182]. Coupling these platforms with artificial intelligence-driven wound management systems could enable predictive modeling of the healing period, automated adjustment of therapy regimens, and remote clinical oversight. Moreover, multimodal therapeutic combinations, such as ROS regulation synergized with immunomodulatory cues and regenerative medicine strategies, may address the multifactorial nature of chronic wounds more effectively than single-modality interventions [183], [184], [185], [186]. These convergent approaches will require interdisciplinary collaboration between materials scientists, bioengineers, clinicians, and data scientists, but hold the promise of transforming ROS-based DDS from experimental concepts into adaptive, patient-specific wound healing solutions capable of dramatically improving quality of life for individuals with recalcitrant wounds.

## 7 Conclusions

ROS-responsive DDS offer unique advantages in chronic wound management by exploiting the pathological hallmark of sustained oxidative stress for site-specific, on-demand therapeutic release. This strategy enables precise spatiotemporal drug deployment, minimizes systemic exposure, and allows integration of multifunctional capabilities, such as antimicrobial action, immunomodulation, and angiogenesis promotion, within a single platform. Moreover, the potential to couple ROS-responsiveness with diagnostic modalities facilitates theranostic applications, enabling real-time monitoring and adaptive treatment. Nevertheless, limitations remain, including the heterogeneity of ROS levels across wound types and stages, possible off-target activation in inflamed but non-wounded tissues, and challenges in achieving long-term stability, scalable manufacturing, and regulatory approval. Overcoming these barriers requires close interdisciplinary collaboration among materials scientists, clinicians, bioengineers, and regulatory experts to refine design parameters, develop standardized evaluation protocols, and conduct rigorous clinical trials. Ultimately, translating ROS-responsive DDS from bench to bedside will depend on harmonizing innovative material science with clinically relevant outcomes, ensuring that therapeutic efficacy, safety, and cost-effectiveness align with the complex needs of chronic wound care.

## Abbreviations

DCFH-DA: 2’,7’-dichlorofluorescein diacetate

DDS: Drug delivery systems

DHE: Dihydroethidium

ECM: Extracellular matrix

EVs: Extracellular vehicles

FA: Fulvic acid

HA: Hyaluronic acid

HBPL: Hyperbranched poly-L-lysine

HIF-1α: Hypoxia-inducible factor-1α

H₂O₂: Hydrogen peroxide

HOCl: Hypochlorous acid

HO-1: Heme oxygenase-1

HPF: Hydroxyphenyl fluorescein

IL-10: Interleukin-10

MOFs: Metal-organic frameworks

MSNs: Mesoporous silica nanoparticles

NADPH: Nicotinamide adenine dinucleotide phosphate

NF-κB: Nuclear factor kappa-B

NIR: Near-infrared

NP: Nanoparticle

Nrf2: Nuclear factor erythroid 2-related factor 2

O₂•⁻: Superoxide anion radical

•OH: hydroxyl radical

ONOO⁻: Peroxynitrite

PBA: Phenylboronic acid

PDGF-B: Platelet-derived growth factor B

PEG: Polyethylene glycol

PG: Pigment prodigiosin

QSI: Quorum-sensing inhibitors

ROS: Reactive oxygen species

VEGF: Vascular endothelial growth factor

## Ethics approval and consent to participate

Not applicable.

## Funding

This work was supported by the National Natural Science Foundation of China (82202714), and the financial support from University Medical Center Groningen (UMCG) and Starterbuers Grant.

## CRediT authorship contribution statement

YX, BBM, and MAS designed the framework. HW, YX, and QPW wrote the manuscript. MAS, YX, and BBM revised the manuscript. All authors read and approved the final version of the manuscript.

## Data Availability

The datasets used or analyzed during the current study are available from the corresponding author on reasonable request.

## References

[1] Sen C.K. (2025). Human wound and its burden: updated 2025 compendium of estimates. Adv Wound Care (New Rochelle).

[2] Graves N., Phillips C.J., Harding K. (2022). A narrative review of the epidemiology and economics of chronic wounds. Br J Dermatol.

[3] Huelsboemer L., Knoedler L., Kochen A., Yu C.T., Hosseini H., Hollmann K.S. (2024). Cellular therapeutics and immunotherapies in wound healing-on the pulse of time?. Mil Med Res.

[4] Martinengo L., Olsson M., Bajpai R., Soljak M., Upton Z., Schmidtchen A. (2019). Prevalence of chronic wounds in the general population: systematic review and meta-analysis of observational studies. Ann Epidemiol.

[5] Sen C.K. (2021). Human wound and its burden: updated 2020 compendium of estimates. Adv Wound Care (New Rochelle).

[6] Xiong Y., Mi B.B., Shahbazi M.A., Xia T., Xiao J. (2024). Microenvironment-responsive nanomedicines: a promising direction for tissue regeneration. Mil Med Res.

[7] Pena O.A., Martin P. (2024). Cellular and molecular mechanisms of skin wound healing. Nat Rev Mol Cell Biol.

[8] Li S., Lu L., Xiong Y., Xiao J. (2025). Nanomedicine-based immunotherapy for tissue regeneration. Burns Trauma.

[9] Ruzicka J., Dejmek J., Bolek L., Benes J., Kuncova J. (2021). Hyperbaric oxygen influences chronic wound healing - a cellular level review. Physiol Res.

[10] Shi R., Zhu Y., Chen Y., Lin Y., Shi S. (2024). Advances in DNA nanotechnology for chronic wound management: Innovative functional nucleic acid nanostructures for overcoming key challenges. J Control Release.

[11] Hunt M., Torres M., Bachar-Wikstrom E., Wikstrom J.D. (2024). Cellular and molecular roles of reactive oxygen species in wound healing. Commun Biol.

[12] Ukaegbu K., Allen E., Svoboda K.K.H. (2025). Reactive oxygen species and antioxidants in wound healing: mechanisms and therapeutic potential. Int Wound J.

[13] Wlaschek M., Singh K., Sindrilaru A., Crisan D., Scharffetter-Kochanek K. (2019). Iron and iron-dependent reactive oxygen species in the regulation of macrophages and fibroblasts in non-healing chronic wounds. Free Radic Biol Med.

[14] Xiong Y., Chu X., Yu T., Knoedler S., Schroeter A., Lu L. (2023). Reactive oxygen species-scavenging nanosystems in the treatment of diabetic wounds. Adv Healthc Mater.

[15] Huang F., Lu X., Kuai L., Ru Y., Jiang J., Song J. (2024). Dual-site biomimetic Cu/Zn-MOF for atopic dermatitis catalytic therapy via suppressing FCγR-mediated phagocytosis. J Am Chem Soc.

[16] Xiong Y., Knoedler S., Alfertshofer M., Kim B.S., Jiang D., Liu G. (2025). Mechanisms and therapeutic opportunities in metabolic aberrations of diabetic wounds: a narrative review. Cell Death Dis.

[17] Deng Q.S., Gao Y., Rui B.Y., Li X.R., Liu P.L., Han Z.Y. (2023). Double-network hydrogel enhanced by SS31-loaded mesoporous polydopamine nanoparticles: symphonic collaboration of near-infrared photothermal antibacterial effect and mitochondrial maintenance for full-thickness wound healing in diabetes mellitus. Bioact Mater.

[18] Zhou W., Xiong P., Ge Y., He Y., Sun Y., Zhang G. (2024). Amoeba-inspired soft robot for integrated tumor/infection therapy and painless postoperative drainage. Adv Sci (Weinh).

[19] Moloney J.N., Cotter T.G. (2018). ROS signalling in the biology of cancer. Semin Cell Dev Biol.

[20] Block M.L., Zecca L., Hong J.S. (2007). Microglia-mediated neurotoxicity: uncovering the molecular mechanisms. Nat Rev Neurosci.

[21] Shah M.S., Brownlee M. (2016). Molecular and cellular mechanisms of cardiovascular disorders in diabetes. Circ Res.

[22] Knoedler S., Knoedler L., Kauke-Navarro M., Rinkevich Y., Hundeshagen G., Harhaus L. (2023). Regulatory T cells in skin regeneration and wound healing. Mil Med Res.

[23] Chang M., Nguyen T.T. (2021). Strategy for treatment of infected diabetic foot ulcers. Acc Chem Res.

[24] Lyons O.T., Saha P., Smith A. (2020). Redox dysregulation in the pathogenesis of chronic venous ulceration. Free Radic Biol Med.

[25] Ruan Y., Cheng J., Dai J., Ma Z., Luo S., Yan R. (2023). Chronic stress hinders sensory axon regeneration via impairing mitochondrial cristae and OXPHOS. Sci Adv.

[26] Bortolotti M., Polito L., Battelli M.G., Bolognesi A. (2021). Xanthine oxidoreductase: one enzyme for multiple physiological tasks. Redox Biol.

[27] Luo X., Xiong H., Jiang Y., Fan Y., Zuo C., Chen D. (2023). Macrophage reprogramming via targeted ROS scavenging and COX-2 downregulation for alleviating inflammation. Bioconjug Chem.

[28] Yang W.S., Kim K.J., Gaschler M.M., Patel M., Shchepinov M.S., Stockwell B.R. (2016). Peroxidation of polyunsaturated fatty acids by lipoxygenases drives ferroptosis. Proc Natl Acad Sci U S A.

[29] Forstermann U., Xia N., Li H. (2017). Roles of vascular oxidative stress and nitric oxide in the pathogenesis of atherosclerosis. Circ Res.

[30] Yu Y.L., Wu J.J., Lin C.C., Qin X., Tay F.R., Miao L. (2023). Elimination of methicillin-resistant Staphylococcus aureus biofilms on titanium implants via photothermally-triggered nitric oxide and immunotherapy for enhanced osseointegration. Mil Med Res.

[31] Chen B., Yang Y., Wang Y., Yan Y., Wang Z., Yin Q. (2021). Precise monitoring of singlet oxygen in specific endocytic organelles by super-pH-resolved nanosensors. ACS Appl Mater Interfaces.

[32] Singh S., Numan A., Khalid M., Bello I., Panza E., Cinti S. (2023). Facile and affordable design of mxene-Co_3_O_4_-based nanocomposites for detection of hydrogen peroxide in cancer cells: toward portable tool for cancer management. Small.

[33] Garcia-Diaz M., Huang Y.Y., Hamblin M.R. (2016). Use of fluorescent probes for ROS to tease apart type I and type II photochemical pathways in photodynamic therapy. Methods.

[34] Sarkar J., Das M., Howlader M.S.I., Prateeksha P., Barthels D., Das H. (2022). Epigallocatechin-3-gallate inhibits osteoclastic differentiation by modulating mitophagy and mitochondrial functions. Cell Death Dis.

[35] Shanmugam G., Narasimhan M., Tamowski S., Darley-Usmar V., Rajasekaran N.S. (2017). Constitutive activation of NRF2 induces a stable reductive state in the mouse myocardium. Redox Biol.

[36] Wang L., Hou X., Fang H., Yang X. (2022). Boronate-based fluorescent probes as a prominent tool for H_2_O_2_ sensing and recognition. Curr Med Chem.

[37] Geng Y., Wang Z., Zhou J., Zhu M., Liu J., James T.D. (2023). Recent progress in the development of fluorescent probes for imaging pathological oxidative stress. Chem Soc Rev.

[38] Huang Y., Liang J., Fan Z. (2023). A review: small organic molecule dual/multi-organelle-targeted fluorescent probes. Talanta.

[39] Perez E., Vazquez L., Quintana C., Petit-Dominguez M.D., Casero E., Blanco E. (2023). Synergistic effect of manganese (II) phosphate & diamond nanoparticles in electrochemical sensors for reactive oxygen species determination in seminal plasma. Anal Chim Acta.

[40] Zhang T., Tian T., Lin Y. (2022). Functionalizing framework nucleic-acid-based nanostructures for biomedical application. Adv Mater.

[41] Wu L., Sedgwick A.C., Sun X., Bull S.D., He X.P., James T.D. (2019). Reaction-based fluorescent probes for the detection and imaging of reactive oxygen, nitrogen, and sulfur species. Acc Chem Res.

[42] Yu X., Ouyang W., Qiu H., Zhang Z., Wang Z., Xing B. (2022). Detection of reactive oxygen and nitrogen species by upconversion nanoparticle-based near-infrared nanoprobes: recent progress and perspectives. Chemistry.

[43] Zhao S., Zang G., Zhang Y., Liu H., Wang N., Cai S. (2021). Recent advances of electrochemical sensors for detecting and monitoring ROS/RNS. Biosens Bioelectron.

[44] Munteanu I.G., Apetrei C. (2021). Analytical methods used in determining antioxidant activity: a review. Int J Mol Sci.

[45] Eruslanov E., Kusmartsev S. (2010). Identification of ROS using oxidized DCFDA and flow-cytometry. Methods Mol Biol.

[46] Miller E.W., Albers A.E., Pralle A., Isacoff E.Y., Chang C.J. (2005). Boronate-based fluorescent probes for imaging cellular hydrogen peroxide. J Am Chem Soc.

[47] Lara-Rojas F., Juarez-Verdayes M.A., Wu H.M., Cheung A.Y., Montiel J., Pascual-Morales E. (2023). Using hyper as a molecular probe to visualize hydrogen peroxide in living plant cells: an updated method. Methods Enzymol.

[48] Kumar R., Gullapalli R.R. (2024). High throughput screening assessment of reactive oxygen species (ROS) generation using dihydroethidium (DHE) fluorescence dye. J Vis Exp.

[49] Kauffman M.E., Kauffman M.K., Traore K., Zhu H., Trush M.A., Jia Z. (2016). MitoSOX-based flow cytometry for detecting mitochondrial ROS. React Oxyg Species (Apex).

[50] Cohn C.A., Pedigo C.E., Hylton S.N., Simon S.R., Schoonen M.A. (2009). Evaluating the use of 3’-(p-Aminophenyl) fluorescein for determining the formation of highly reactive oxygen species in particle suspensions. Geochem Trans.

[51] Sugimoto W., Miyoshi D., Kawauchi K. (2021). Detection of intracellular reactive oxidative species using the fluorescent probe hydroxyphenyl fluorescein. Methods Mol Biol.

[52] Hachfi S., Benguettat O., Gallet A. (2019). Hypochlorous acid staining with R19-S in the Drosophila intestine upon ingestion of opportunistic bacteria. Bio Protoc.

[53] Song Z.G., Yuan Q., Lv P., Chen K. (2021). Research progress of small molecule fluorescent probes for detecting hypochlorite. Sensors (Basel).

[54] Zhang Y., Liu D., Chen W., Tao Y., Li W., Qi J. (2024). Microenvironment-activatable probe for precise NIR-II monitoring and synergistic immunotherapy in rheumatoid arthritis. Adv Mater.

[55] Murphy M.P., Bayir H., Belousov V., Chang C.J., Davies K.J.A., Davies M.J. (2022). Guidelines for measuring reactive oxygen species and oxidative damage in cells and in vivo. Nat Metab.

[56] Zhang W., Hao L., Huang J., Xia L., Cui M., Zhang X. (2019). Chemiluminescence chitosan hydrogels based on the luminol analog L-012 for highly sensitive detection of ROS. Talanta.

[57] Kim H., An H.J., Park J., Lee Y., Kim M.S., Lee S. (2022). Ultrasensitive and real-time optical detection of cellular oxidative stress using graphene-covered tunable plasmonic interfaces. Nano Converg.

[58] Olson K.R., Gao Y., Arif F., Arora K., Patel S., DeLeon E.R. (2018). Metabolism of hydrogen sulfide (H_2_S) and production of reactive sulfur species (RSS) by superoxide dismutase. Redox Biol.

[59] Shen J., Shi W., Liu G., Zhuang W., Wang K., Wang Y. (2023). Early diagnosis and treatment of osteoarthritis with a Au@PDA-WL NP nano-probe by photoacoustic imaging. J Mater Chem B.

[60] Ge C., Zhu J., Wu G., Ye H., Lu H., Yin L. (2022). ROS-responsive selenopolypeptide micelles: preparation, characterization, and controlled drug release. Biomacromolecules.

[61] Li J., Du Y., Wang J., Liu T., Zhu H., Ma J. (2025). Optimizing type H vessels formation via short fibers 3D scaffolds with maintaining redox homeostasis for osteoporotic bone remodeling. Bioact Mater.

[62] Lu Z., Chai Q., Dai W., Yu B., Lv Q., Qiu F. (2025). Mitochondrial homeostasis restoring peptide-drug conjugates with ROS-responsive NO releasing ability for targeted therapy of myocardial infarction. J Nanobiotechnology.

[63] Zhu Y., Chang B., Pang Y., Wang H., Zhou Y. (2023). Advances in hypoxia-inducible factor-1α stabilizer deferoxamine in tissue engineering. Tissue Eng Part B Rev.

[64] Jin L., Zhu Z., Hong L., Qian Z., Wang F., Mao Z. (2023). ROS-responsive 18β-glycyrrhetic acid-conjugated polymeric nanoparticles mediate neuroprotection in ischemic stroke through HMGB1 inhibition and microglia polarization regulation. Bioact Mater.

[65] Li H., Li Y., Zhang L., Wang N., Lu D., Tang D. (2024). Prodrug-inspired adenosine triphosphate-activatable celastrol-Fe(III) chelate for cancer therapy. Sci Adv.

[66] Cheng H., Shi Z., Yue K., Huang X., Xu Y., Gao C. (2021). Sprayable hydrogel dressing accelerates wound healing with combined reactive oxygen species-scavenging and antibacterial abilities. Acta Biomater.

[67] Zhang S.J., Xu R., He S.B., Sun R., Wang G.N., Wei S.Y. (2025). Nanozyme-driven multifunctional dressings: moving beyond enzyme-like catalysis in chronic wound treatment. Mil Med Res.

[68] Tao L., Ma X., Yang Y., Wang H., Yang X., Luo Y. (2025). Continuous pressure exacerbates ischemia-reperfusion injury in minipigs through the Akt/eNOS signaling pathway. Eur J Med Res.

[69] Qu J., Zhao X., Liang Y., Zhang T., Ma P.X., Guo B. (2018). Antibacterial adhesive injectable hydrogels with rapid self-healing, extensibility and compressibility as wound dressing for joints skin wound healing. Biomaterials.

[70] Chen Y., Wang X., Tao S., Wang Q., Ma P.Q., Li Z.B. (2023). Research advances in smart responsive-hydrogel dressings with potential clinical diabetic wound healing properties. Mil Med Res.

[71] Wang G., Yang F., Zhou W., Xiao N., Luo M., Tang Z. (2023). The initiation of oxidative stress and therapeutic strategies in wound healing. Biomed Pharmacother.

[72] Li G., Ko C.N., Li D., Yang C., Wang W., Yang G.J. (2021). A small molecule HIF-1alpha stabilizer that accelerates diabetic wound healing. Nat Commun.

[73] Li H., Yuan Y., Zhang L., Xu C., Xu H., Chen Z. (2024). Reprogramming macrophage polarization, depleting ROS by astaxanthin and thioketal-containing polymers delivering rapamycin for osteoarthritis treatment. Adv Sci (Weinh).

[74] Pei P., Sun C., Tao W., Li J., Yang X., Wang J. (2019). ROS-sensitive thioketal-linked polyphosphoester-doxorubicin conjugate for precise phototriggered locoregional chemotherapy. Biomaterials.

[75] Criado-Gonzalez M., Mecerreyes D. (2022). Thioether-based ROS responsive polymers for biomedical applications. J Mater Chem B.

[76] Cao W., Gu Y., Li T., Xu H. (2015). Ultra-sensitive ROS-responsive tellurium-containing polymers. Chem Commun (Camb).

[77] Gao J., Zhai Y., Lu W., Jiang X., Zhou J., Wu L. (2024). ROS-sensitive PD-L1 siRNA cationic selenide nanogels for self-inhibition of autophagy and prevention of immune escape. Bioact Mater.

[78] Yang H., Wang W., Xiao J., Yang R., Feng L., Xu H. (2025). ROS-responsive injectable hydrogels loaded with exosomes carrying miR-4500 reverse liver fibrosis. Biomaterials.

[79] Kim K.S., Lee D., Song C.G., Kang P.M. (2015). Reactive oxygen species-activated nanomaterials as theranostic agents. Nanomedicine (Lond).

[80] Sheng Y., Zhang C., Cai D., Xu G., Chen S., Li W. (2024). 2,2’,4,4’-Tetrabromodiphenyl ether and cadmium co-exposure activates aryl hydrocarbon receptor pathway to induce ROS and GSDME-dependent pyroptosis in renal tubular epithelial cells. Environ Toxicol.

[81] Shi G., Wu Z., Hao Z., Zhu M., Shu F., Yang Z. (2025). Microenvironment-responsive hydrogels comprising engineering zeolitic imidazolate framework-8-anchored parathyroid hormone-related peptide-1 for osteoarthritis therapy. ACS Nano.

[82] Yan J., Wang Y., Zhang J., Liu X., Yu L., He Z. (2023). Rapidly blocking the calcium overload/ROS production feedback loop to alleviate acute kidney injury via microenvironment-responsive BAPTA-AM/BAC co-delivery nanosystem. Small.

[83] Zhang Y., Zhou X., Liang G., Cui M., Qiu Z., Xu J. (2025). Iron-chelating and ROS-scavenging polymers with thioketal and thioether bonds delivering ferroptosis inhibitor lip-1 provide a triple therapeutic strategy for retina ganglion cells in acute glaucoma. Adv Mater.

[84] Liu L., Wang W., Huang L., Xian Y., Ma W., Fan J. (2024). Injectable pathological microenvironment-responsive anti-inflammatory hydrogels for ameliorating intervertebral disc degeneration. Biomaterials.

[85] Hu D., Li Y., Li R., Wang M., Zhou K., He C. (2024). Recent advances in reactive oxygen species (ROS)-responsive drug delivery systems for photodynamic therapy of cancer. Acta Pharm Sin B.

[86] Fan K., Yang D., Zhu X., Zheng L., Han Y., Lin J. (2025). High-efficiency antioxidant ROS-responsive thermosensitive hydrogel encapsulated Fenofibrate for the treatment of corneal neovascularization. J Control Release.

[87] Yu Y., Jiang X., Yu T., Chen F., Huang R., Xun Z. (2025). Maintaining myoprotein and redox homeostasis via an orally recharged nanoparticulate supplement potentiates sarcopenia treatment. Biomaterials.

[88] Ahmed W., Li S., Liang M., Kang Y., Liu X., Gao C. (2024). Multifunctional drug- and AuNRs-loaded ROS-responsive selenium-containing polyurethane nanofibers for smart wound healing. ACS Biomater Sci Eng.

[89] Gao Y., Yang R., Shou Z., Zan X., Tang S. (2024). Optimization of boronic ester-based amphiphilic copolymers for ROS-responsive drug delivery. Chem Commun (Camb).

[90] Song F., Li S., Sun C., Ji Y., Zhang Y. (2021). ROS-responsive selenium-containing carriers for coencapsulation of photosensitizer and hypoxia-activated prodrug and their cellular behaviors. Macromol Biosci.

[91] Zhang Y., Liang Z., Tan Z., Lin J., Jiang X., Zhou X. (2025). Bioinformatics-driven engineering of ROS-responsive dextran-block-poly(propylene sulfide) nanoparticles functionalized with folic acid for targeted prostate cancer therapy. ACS Omega.

[92] Xiong Y., Feng Q., Lu L., Qiu X., Knoedler S., Panayi A.C. (2024). Metal-organic frameworks and their composites for chronic wound healing: from bench to bedside. Adv Mater.

[93] Wu Y., Wang Y., Long L., Hu C., Kong Q., Wang Y. (2022). A spatiotemporal release platform based on pH/ROS stimuli-responsive hydrogel in wound repairing. J Control Release.

[94] Huang T., Yuan B., Jiang W., Ding Y., Jiang L., Ren H. (2021). Glucose oxidase and Fe_3_O_4_/TiO_2_/Ag_3_PO_4_ co-embedded biomimetic mineralization hydrogels as controllable ROS generators for accelerating diabetic wound healing. J Mater Chem B.

[95] Jeong S.H., Cheong S., Kim T.Y., Choi H., Hahn S.K. (2023). Supramolecular hydrogels for precisely controlled antimicrobial peptide delivery for diabetic wound healing. ACS Appl Mater Interfaces.

[96] Guo C., Wu Y., Li W., Wang Y., Kong Q. (2022). Development of a microenvironment-responsive hydrogel promoting chronically infected diabetic wound healing through sequential hemostatic, antibacterial, and angiogenic activities. ACS Appl Mater Interfaces.

[97] Shi R., Li H., Jin X., Huang X., Ou Z., Zhang X. (2022). Promoting Re-epithelialization in an oxidative diabetic wound microenvironment using self-assembly of a ROS-responsive polymer and P311 peptide micelles. Acta Biomater.

[98] Ma W., Zhang X., Liu Y., Fan L., Gan J., Liu W. (2022). Polydopamine decorated microneedles with Fe-MSC-derived nanovesicles encapsulation for wound healing. Adv Sci (Weinh).

[99] Zhao M., Kang M., Wang J., Yang R., Zhong X., Xie Q. (2024). Stem cell-derived nanovesicles embedded in dual-layered hydrogel for programmed ROS regulation and comprehensive tissue regeneration in burn wound healing. Adv Mater.

[100] Yang H., Lv D., Qu S., Xu H., Li S., Wang Z. (2024). A ROS-responsive lipid nanoparticles release multifunctional hydrogel based on microenvironment regulation promotes infected diabetic wound healing. Adv Sci (Weinh).

[101] Chen Y., Lei K., Li Y., Mu Z., Chu T., Hu J. (2025). Synergistic effects of NO/H_2_S gases on antibacterial, anti-inflammatory, and analgesic properties in oral ulcers using a gas-releasing nanoplatform. Acta Biomater.

[102] Tu C., Lu H., Zhou T., Zhang W., Deng L., Cao W. (2022). Promoting the healing of infected diabetic wound by an anti-bacterial and nano-enzyme-containing hydrogel with inflammation-suppressing, ROS-scavenging, oxygen and nitric oxide-generating properties. Biomaterials.

[103] Tian S., Mei J., Zhang L., Wang S., Yuan Y., Li J. (2024). Multifunctional hydrogel microneedle patches modulating oxi-inflamm-aging for diabetic wound healing. Small.

[104] Xiao J., An X., Tang F., Dai X., Zhang S., Zhu X. (2025). Photosynthesis-inspired NIR-triggered Fe_3_O_4_@MoS_2_ core-shell nanozyme for promoting MRSA-infected diabetic wound healing. Adv Healthc Mater.

[105] Xiong Y., Chen L., Liu P., Yu T., Lin C., Yan C. (2022). All-in-one: multifunctional hydrogel accelerates oxidative diabetic wound healing through timed-release of exosome and fibroblast growth factor. Small.

[106] Wu L., Lu Y., Liu L., Wang J., Bai Y., Song J. (2024). BaTiO_3_ doping enhances ultrasound-driven piezoelectric bactericidal effects of fibrous poly(L-lactic acid) dressings to accelerate septic wound healing. ACS Appl Mater Interfaces.

[107] Huang Z.J., Ye M.N., Peng X.H., Gui P., Cheng F., Wang G.H. (2025). Thiolated chitosan hydrogel combining nitric oxide and silver nanoparticles for the effective treatment of diabetic wound healing. Int J Biol Macromol.

[108] Puertas-Bartolome M., Wlodarczyk-Biegun M.K., Del Campo A., Vazquez-Lasa B., San Roman J. (2021). Development of bioactive catechol functionalized nanoparticles applicable for 3D bioprinting. Mater Sci Eng C Mater Biol Appl.

[109] Wu S., Zhang H., Wang S., Sun J., Hu Y., Liu H. (2023). Ultrasound-triggered in situ gelation with ROS-controlled drug release for cartilage repair. Mater Horiz.

[110] Elbedwehy A.M., Wu J., Na H.K., Baek A., Jung H., Kwon I.H. (2024). ROS-responsive charge reversal mesoporous silica nanoparticles as promising drug delivery system for neovascular retinal diseases. J Control Release.

[111] Li X., Li Y., Yu C., Bao H., Cheng S., Huang J. (2023). ROS-responsive janus Au/mesoporous silica core/shell nanoparticles for drug delivery and long-term CT imaging tracking of MSCs in pulmonary fibrosis treatment. ACS Nano.

[112] Xiong Y., Mi B., Liu G., Zhao Y. (2024). Microenvironment-sensitive nanozymes for tissue regeneration. Biomaterials.

[113] Luo L.J., Nguyen D.D., Lai J.Y. (2021). Harnessing the tunable cavity of nanoceria for enhancing Y-27632-mediated alleviation of ocular hypertension. Theranostics.

[114] Yang C.J., Nguyen D.D., Lai J.Y. (2023). Poly(l-histidine)-mediated on-demand therapeutic delivery of roughened ceria nanocages for treatment of chemical eye injury. Adv Sci (Weinh).

[115] Li M., Tian J., Yu K., Liu H., Yu X., Wang N. (2024). A ROS-responsive hydrogel incorporated with dental follicle stem cell-derived small extracellular vesicles promotes dental pulp repair by ameliorating oxidative stress. Bioact Mater.

[116] Nguyen D.D., Lai J.Y. (2020). Advancing the stimuli response of polymer-based drug delivery systems for ocular disease treatment. Polymer Chemistry.

[117] Nguyen D.D., Lai J.Y. (2022). Synthesis, bioactive properties, and biomedical applications of intrinsically therapeutic nanoparticles for disease treatment. Chem Eng J.

[118] Geng C., He S., Yu S., Johnson H.M., Shi H., Chen Y. (2024). Achieving clearance of drug-resistant bacterial infection and rapid cutaneous wound regeneration using an ROS-balancing-engineered heterojunction. Adv Mater.

[119] Gu C., Fang S., Liu L., Chen B., Xu L., Shao M. (2024). Local release of copper manganese oxide using HA microneedle for improving the efficacy of drug-resistant wound inflammation. Small.

[120] Kang Y., Xu L., Dong J., Yuan X., Ye J., Fan Y. (2024). Programmed microalgae-gel promotes chronic wound healing in diabetes. Nat Commun.

[121] Xiong R., Zhou M., Li H., Wang L., Ling G., Zhang P. (2025). The three-pronged strategy: a bilayer hydrogel treats diabetic chronic wound through microalgae oxygen therapy, Ag horizontal line FA NP antibacterial, and synergistic scavenging of ROS. Small.

[122] Zhang X., Ren K., Xiao C., Chen X. (2023). Guanosine-driven hyaluronic acid-based supramolecular hydrogels with peroxidase-like activity for chronic diabetic wound treatment. Acta Biomater.

[123] Pang Y., Amona F.M., Chen X., You Y., Sha Z., Liu Z. (2025). Phytochemical nanozymes reprogram redox for balanced antimicrobial and regenerative therapy in acute and chronic diabetic wounds. Redox Biol.

[124] Zhao A., Sun J., Liu Y. (2023). Understanding bacterial biofilms: from definition to treatment strategies. Front Cell Infect Microbiol.

[125] Liu G.Y., Yu D., Fan M.M., Zhang X., Jin Z.Y., Tang C. (2024). Antimicrobial resistance crisis: could artificial intelligence be the solution?. Mil Med Res.

[126] Li Y.J., Wei S.C., Chu H.W., Jian H.J., Anand A., Nain A. (2022). Poly-quercetin-based nanoVelcro as a multifunctional wound dressing for effective treatment of chronic wound infections. Chem Eng J.

[127] Zha K., Xiong Y., Zhang W., Tan M., Hu W., Lin Z. (2023). Waste to wealth: near-infrared/pH dual-responsive copper-humic acid hydrogel films for bacteria-infected cutaneous wound healing. ACS Nano.

[128] Li J., Wang M., Tan X., Duanmiao Y., Zheng X., Wang Z. (2025). A dual-component particulate dressing for simultaneous microenvironment modulation and tissue regeneration in infected diabetic wounds. Mater Today Bio.

[129] Li Y., Wang Y., Ding Y., Fan X., Ye L., Pan Q. (2024). A double network composite hydrogel with self-regulating Cu^2+^/luteolin release and mechanical modulation for enhanced wound healing. ACS Nano.

[130] Nie R., Zhang J., Jia Q., Li Y., Tao W., Qin G. (2024). Structurally oriented carbon dots as ROS nanomodulators for dynamic chronic inflammation and infection elimination. ACS Nano.

[131] Zhang W., Zha K., Xiong Y., Hu W., Chen L., Lin Z. (2023). Glucose-responsive, antioxidative HA-PBA-FA/EN106 hydrogel enhanced diabetic wound healing through modulation of FEM1b-FNIP1 axis and promoting angiogenesis. Bioact Mater.

[132] Zhang Z., Zhang Y., Peng L., Xing Y., Zhou X., Zheng S. (2025). Multifunctional dual-layer microneedles loaded with selenium-doped carbon quantum dots and Astilbin for ameliorating diabetic wound healing. Mater Today Bio.

[133] Li H., Wen H., Zhang H., Cao X., Li L., Hu X. (2025). A multifunctional dihydromyricetin-loaded hydrogel for the sequential modulation of diabetic wound healing and glycemic control. Burns Trauma.

[134] Huang K., Mi B., Xiong Y., Fu Z., Zhou W., Liu W. (2025). Angiogenesis during diabetic wound repair: from mechanism to therapy opportunity. Burns Trauma.

[135] Kuan C.H., Chang L., Ho C.Y., Tsai C.H., Liu Y.C., Huang W.Y. (2025). Immunomodulatory hydrogel orchestrates pro-regenerative response of macrophages and angiogenesis for chronic wound healing. Biomaterials.

[136] He F., Xu P., Zhu Z., Zhang Y., Cai C., Zhang Y. (2025). Inflammation-responsive hydrogel accelerates diabetic wound healing through immunoregulation and enhanced angiogenesis. Adv Healthc Mater.

[137] Wang M., Chen J., Luo Y., Feng M., Yang Q., Tang Y. (2024). Design strategies and application potential of multifunctional hydrogels for promoting angiogenesis. Int J Nanomedicine.

[138] Xiang P., Jiang M., Chen X., Chen L., Cheng Y., Luo X. (2024). Targeting grancalcin accelerates wound healing by improving angiogenesis in diabetes. Adv Sci (Weinh).

[139] Guan Y., Niu H., Liu Z., Dang Y., Shen J., Zayed M. (2021). Sustained oxygenation accelerates diabetic wound healing by promoting epithelialization and angiogenesis and decreasing inflammation. Sci Adv.

[140] Wang J., Li X., Zhao X., Yuan S., Dou H., Cheng T. (2024). *Lactobacillus rhamnosus* GG-derived extracellular vesicles promote wound healing via miR-21-5p-mediated re-epithelization and angiogenesis. J Nanobiotechnology.

[141] Wang K., He Q., Yang M., Qiao Q., Chen J., Song J. (2024). Glycoengineered extracellular vesicles released from antibacterial hydrogel facilitate diabetic wound healing by promoting angiogenesis. J Extracell Vesicles.

[142] Zeng R., Xiong Y., Lin Z., Chu X., Lv B., Lu L. (2024). Novel cocktail therapy based on multifunctional supramolecular hydrogel targeting immune-angiogenesis-nerve network for enhanced diabetic wound healing. J Nanobiotechnology.

[143] Chen S., Chen J., Wang X., Yang Z., Lan J., Wang L. (2025). Glucose-activated self-cascade antibacterial and pro-angiogenesis nanozyme-functionalized chitosan-arginine thermosensitive hydrogel for chronic diabetic wounds healing. Carbohydr Polym.

[144] Li F., Du Y., Zheng Y., Liu Y., Zhu X., Cui Y. (2025). Microenvironment-responsive MOF nanozymes armored cryogels promoted wound healing via rapid hemostasis, infection elimination and angiogenesis. J Control Release.

[145] Tian X., Wen Y., Zhang Z., Zhou K., Shang L., Zhu J. (2026). Smart multifunctional ROS-responsive supramolecular hydrogel for simultaneously regulating oxidative stress, immune dysregulation, and bacterial infection in diabetic wound healing. Biomaterials.

[146] Huang K., Liu W., Wei W., Zhao Y., Zhuang P., Wang X. (2022). Photothermal hydrogel encapsulating intelligently bacteria-capturing Bio-MOF for infectious wound healing. ACS Nano.

[147] Liu W.S., Lu Z.M., Pu X.H., Li X.Y., Zhang H.Q., Zhang Z.Z. (2025). A dendritic cell-recruiting, antimicrobial blood clot hydrogel for melanoma recurrence prevention and infected wound management. Biomaterials.

[148] Wu Z., Chang L., Li C., Xu P., Liu L., Tong A. (2025). Prodigiosin loaded SN-PB@PG NPs-based multimodal therapy for the healing of bacterial infected chronic wounds. Adv Healthc Mater.

[149] Zhang R., Wang S., Ma X., Jiang S., Chen T., Du Y. (2022). situ gelation strategy based on ferrocene-hyaluronic acid organic copolymer biomaterial for exudate management and multi-modal wound healing. Acta Biomater.

[150] Li H., Feng Y., Lin B., Zhang S., Ren Y., Yue J. (2026). Polyurea-based multimodal interaction nanogels for synergistic bacterial biofilm eradication and prevention of re-colonization. Biomaterials.

[151] Wu J., Wu Y., Tang H., Li W., Zhao Z., Shi X. (2024). Self-adapting biomass hydrogel embodied with miRNA immunoregulation and long-term bacterial eradiation for synergistic chronic wound therapy. ACS Nano.

[152] Liu J., Han X., Zhang T., Tian K., Li Z., Luo F. (2023). Reactive oxygen species (ROS) scavenging biomaterials for anti-inflammatory diseases: from mechanism to therapy. J Hematol Oncol.

[153] Cao W., Peng S., Yao Y., Xie J., Li S., Tu C. (2022). A nanofibrous membrane loaded with doxycycline and printed with conductive hydrogel strips promotes diabetic wound healing in vivo. Acta Biomater.

[154] Shen J., Tong Z., Han B., Zhang Z., Xian Z., Yuan Y. (2025). Synergistic wound healing: unraveling the multi-target effects of traditional Chinese medicine and its biomaterials on chronic wound pathways. Int J Nanomedicine.

[155] Horta-Velazquez A., Mota-Morales J.D., Morales-Narvaez E. (2024). Next-generation of smart dressings: integrating multiplexed sensors and theranostic functions. Int J Biol Macromol.

[156] Burkett B.J., Bartlett D.J., McGarrah P.W., Lewis A.R., Johnson D.R., Berberoglu K. (2023). A review of theranostics: perspectives on emerging approaches and clinical advancements. Radiol Imaging Cancer.

[157] Duan W., Zhao J., Liu X., Zheng Y., Wu J. (2023). Trapping and release of NIR-active dye in porous silicon as a theranostic strategy for ROS photothermal monitoring and chronic wound management. J Control Release.

[158] Joorabloo A., Liu T. (2024). Smart theranostics for wound monitoring and therapy. Adv Colloid Interface Sci.

[159] Chen Y., Yang X., Li K., Feng J., Liu X., Li Y. (2024). Phenolic ligand-metal charge transfer induced copper nanozyme with reactive oxygen species-scavenging ability for chronic wound healing. ACS Nano.

[160] Zhao Y., Zhao Y., Xu B., Liu H., Chang Q. (2024). Microenvironmental dynamics of diabetic wounds and insights for hydrogel-based therapeutics. J Tissue Eng.

[161] Wang S., Zhang Y., Zhong Y., Xue Y., Liu Z., Wang C. (2024). Accelerating diabetic wound healing by ROS-scavenging lipid nanoparticle-mRNA formulation. Proc Natl Acad Sci U S A.

[162] Gumede D.B., Abrahamse H., Houreld N.N. (2024). Targeting Wnt/β-catenin signaling and its interplay with TGF-β and Notch signaling pathways for the treatment of chronic wounds. Cell Commun Signal.

[163] Stewart D.C., Brisson B.K., Yen W.K., Liu Y., Wang C., Ruthel G. (2025). Type III collagen regulates matrix architecture and mechanosensing during wound healing. J Invest Dermatol.

[164] He J., Cheng X., Fang B., Shan S., Li Q. (2024). Mechanical stiffness promotes skin fibrosis via Piezo1-Wnt2/Wnt11-CCL24 positive feedback loop. Cell Death Dis.

[165] Patil P., Russo K.A., McCune J.T., Pollins A.C., Cottam M.A., Dollinger B.R. (2022). Reactive oxygen species-degradable polythioketal urethane foam dressings to promote porcine skin wound repair. Sci Transl Med.

[166] Sabio L., Day G.J., Salmeron-Sanchez M. (2026). Probiotic-based materials as living therapeutics. Adv Mater.

[167] Alam A., Kumar A., Jiji S., Akhila K., Khandelwal M. (2025). Integrating life into material design for living materials 4.0: navigating challenges and future trajectories from static to dynamic evolution. Materials Today.

[168] Liu Y., Yang M., Li Y., Liu Y., Su H., Zhang W. (2025). A multifunctional living hydrogel for the synergistic management of infected diabetic wounds. Mater Today Bio.

[169] Toprak B., Kalaycioglu G.D., Aydogan N. (2025). Multifunctional CD-MOF hybrid systems: integrating drug delivery, photothermal therapy, and nanozyme applications. Small.

[170] Yayun J., Qianqian G., Xihang S., Yang H., Zhenping H., Yuying L. (2025). Metal-organic framework-mediated antioxidant enzyme delivery in disease treatment. Redox Biol.

[171] Cheng Q., Cheng C., Xu L. (2025). Polysaccharide-silane polymer composites: a novel electrically tunable drug delivery platform for polycystic ovary syndrome treatment. Carbohydr Res.

[172] Tong M., Kuang X., Jiang Q., Li G., Jin L., Ye Y. (2025). Spore-inspired inhalation drug delivery system for asthma therapy. Bioact Mater.

[173] Wang Y., Sun D., Laney V., Wang H., Wang L.L., Lu Z.R. (2025). Challenges and opportunities on achieving an adequate delivery efficiency and immunogenicity with peptide-based anticancer vaccines. Adv Drug Deliv Rev.

[174] Cheng P., Xie X., Hu L., Zhou W., Mi B., Xiong Y. (2024). Hypoxia endothelial cells-derived exosomes facilitate diabetic wound healing through improving endothelial cell function and promoting M2 macrophages polarization. Bioact Mater.

[175] Li Z., Mao K., Yue M., Liu Y., Fan K. (2025). Nanozymes in oral medicine: from catalytic design to advanced dental therapies. Small.

[176] Yang Z., Qin R., Ruan D., Hu C., Li W., Zhou J. (2025). Ce6-DNAzyme-loaded metal-organic framework theranostic agents for boosting miRNA imaging-guided photodynamic therapy in breast cancer. ACS Nano.

[177] Wang Z., Zeng X., Feng W., Lu Y., Wei P. (2025). Reframing chronic wound therapy: from growth factor delivery to regenerative immuno-engineering. Front Immunol.

[178] Saeed S., Martins-Green M. (2024). Assessing animal models to study impaired and chronic wounds. Int J Mol Sci.

[179] Lou J., Xiang Z., Zhu X., Li J., Jin G., Cui S. (2025). Skin microbiota and diabetic foot ulcers. Front Microbiol.

[180] Chen M., Liu D., Liu F., Wu Y., Peng X., Song F. (2021). Recent advances of redox-responsive nanoplatforms for tumor theranostics. J Control Release.

[181] Li Q., Li J., Song S., Chen W., Shen X., Li S. (2021). Nanoparticle-mediated tumor vaccines for personalized therapy: preparing tumor antigens in vivo or ex vivo?. J Mater Chem B.

[182] Wang Y., Su P., Lin Z., Li X., Chen K., Ye T. (2025). A tribo/piezoelectric nanogenerator based on Bio-MOFs for energy harvesting and antibacterial wearable device. Adv Mater.

[183] Gong J., Ye C., Ran J., Xiong X., Fang X., Zhou X. (2023). Polydopamine-mediated immunomodulatory patch for diabetic periodontal tissue regeneration assisted by metformin-ZIF system. ACS Nano.

[184] Song J., Cheng M., Xie Y., Li K., Zang X. (2023). Efficient tumor synergistic chemoimmunotherapy by self-augmented ROS-responsive immunomodulatory polymeric nanodrug. J Nanobiotechnology.

[185] Yang L., Zhang D., Li W., Lin H., Ding C., Liu Q. (2023). Biofilm microenvironment triggered self-enhancing photodynamic immunomodulatory microneedle for diabetic wound therapy. Nat Commun.

[186] Zhou H., Liao Y., Han X., Chen D.S., Hong X., Zhou K. (2023). ROS-responsive nanoparticle delivery of mRNA and photosensitizer for combinatorial cancer therapy. Nano Lett.

